# Quantification profiles of enzyme activity, secretion, and psychosine levels of Krabbe disease galactosylceramidase missense variants

**DOI:** 10.1016/j.jbc.2025.110315

**Published:** 2025-05-29

**Authors:** Hui Peng, Ying-Wai Lam, Kwok-Fai Lau, Zitao Zhou, Aimee R. Herdt, Michael H. Gelb, Chris W. Lee

**Affiliations:** 1 Biomedical Research Institute of New Jersey (BRInj), Cedar Knolls, New Jersey, USA; 2 Atlantic Health System, Morristown, New Jersey, USA; 3 MidAtlantic Neonatology Associates (MANA), Morristown, New Jersey, USA; 4 Department of Biology & Vermont Biomedical Research Network Proteomics Facility, University of Vermont, Burlington, Vermont, USA; 5 School of Life Sciences, The Chinese University of Hong Kong, Hong Kong SAR, China; 6 Departments of Chemistry and Biochemistry, University of Washington, Seattle, Washington, USA

**Keywords:** Krabbe disease, galactosylceramidase, psychosine, genotype-phenotype correlation, missense mutation variant, pathogenic variant expression study, protein mistrafficking, lysosomal storage disorder

## Abstract

Krabbe disease is an autosomal recessive, demyelinating disorder caused by mutations in the *GALC* gene. Missense mutation variants (MMVs) account for most pathogenic alleles in patients; however, their mechanistic implications and correlations to clinical phenotype remain unclear. To address these questions, we generated a *GALC* knockout human oligodendrocytic cell line to conduct a robust *GALC*-MMVs expression study using a panel of 31 GALC-MMVs. Twenty-six clinically relevant variants dramatically reduced enzyme activity (92–100%). Notably, residual GALC activity strongly correlated with the age of disease-onset in reported cases (Pearson’s r > 0.94, *p* < 0.0001), suggesting that enzyme activity resulting from MMV expression in this model may serve as a readout for clinical prognostication. In addition, we identified p.I562T, a predominant pseudodeficiency variant in the newborn screening programs, which synergistically impairs protein function and likely triggers disease-onset when inherited co-allelic with certain MMVs. We also identified MMVs that increased protein retention intracellularly and/or decreased secretion. This quantitative analysis of misfolding characteristics could be valuable for identifying MMVs amenable to pharmacological chaperone therapy. Finally, we observed an inverse correlation between residual GALC activity and endogenous psychosine levels in the MMV panel. Given the importance of psychosine as a biomarker for diagnosis and newborn screening, the psychosine accumulation phenotype in our model highlights its potential use for drug discovery. Overall, this study provides a comprehensive overview of the functional deficits and mis-trafficking caused by GALC-MMVs, deepens our understanding of molecular genetics and genotype-phenotype correlations in Krabbe disease, and highlights the potential of our platform for genetic and therapeutic applications.

Globoid cell leukodystrophy or Krabbe disease (KD) (OMIM 245200) is an inherited demyelinating disorder caused by a deficiency of galactosylceramidase (GALC; EC 3.2.1.46). GALC function is essential for myelin metabolism (1, 2, 3, 4, 5). The loss of GALC activity leads to the accumulation of psychosine, as well as the dysfunction and death of oligodendrocytes and Schwann cells; thus leading to demyelination in both the central and peripheral nervous systems (6, 7, 8, 9).

According to the American College of Medical Genetics and Genomics guidelines, 254 KD-related mutations in the *GALC* gene have been classified as pathogenic (ClinVar) (10). Among those, 47 are missense mutation variants (MMVs). Statistics from over 20 years of published studies document KD patients from different geographical regions with varying disease severity and show that the majority of patients carried at least one *GALC* MMV allele (Table 1) (11, 12, 13, 14, 15, 16, 17, 18). In the Bascou *et al.* study, 19 of 20 patients had at least one *GALC* MMV allele and 12 had infantile-onset KD (12). These collective observations highlight that *GALC* MMVs are common in the KD population.

**Table 1:** Frequency of *GALC* missense mutation allele in KD population

Study	KD patients		MM allele	Age-of-onset		Patient’s geographic
	Case	MM allele	Frequency	Range (month)	Median (month)	
Madsen *et al.* 2019 (11)	29	11	38%	1–36	3.3	Denmark
Bascou *et al.* 2018 (12)	20	19	95%	6–36	9	United States
Duffner *et al.* 2012 (13)	14	13	93%	8–384	15	European
Duffner *et al.* 2011 (14)	16	7	44%	2–5	3	European
Tappino *et al.* 2010 (15)	30	21	70%	1–312	5	Italian
Xu *et al.* 2006 (16)	14	12	86%	4–828	16	Japan
Fu *et al.* 1999 (17)	8	6	75%	Infantile	N.A.	Japan
De Gasperi *et al.* 1996 (18)	10	8	80%	5–216	54	United States

KD is an autosomal recessive disorder. Most KD patients are compound heterozygous for *GALC* mutations. Patients of northern European descent most frequently carry a 30 kb deletion on one allele (19, 20) and a MMV on the other allele (13, 14). Null mutations, such as the 30 kb deletion, eliminate normal *GALC* transcripts and GALC protein, leading to complete functional deficiency. By contrast, MMVs impair GALC function *via* alternative mechanisms, such as protein misfolding, instability, mis-trafficking, and catalytic inactivation.

GALC is a glycoprotein that is transported to the lysosome *via* the mannose 6-phosphate receptor–mediated pathway (21). Briefly, GALC is modified posttranslationally in the Golgi. Precursor GALC (pre-GALC) is then secreted out of the cell by secretory vesicles to become secreted GALC (sec-GALC) or transported to lysosomes *via* the endosomal pathway (Fig. 1*A*). Of note, upon delivery to the lysosome, GALC undergoes a unique proteolytic cleavage event that generates amino-terminal (∼50 kDa) and carboxyl terminal (C-terminal, ∼30 KDa) fragments (22), held together by disulfide bridges (23). Concomitantly, the extreme C-terminal end of the 30 kDa fragment is proteolytically trimmed further, rendering the fragment undetectable using antibodies against protein tags fused to the C-terminal end (24, 25) (Fig. 1*B*). Although the functional effects of the proteolytic cleavages have not been determined, we expect levels of cleaved GALC fragment (lys-GALC) to be proportional to the mature, active form of GALC in lysosomes (24, 25, 26).

**Figure 1:**
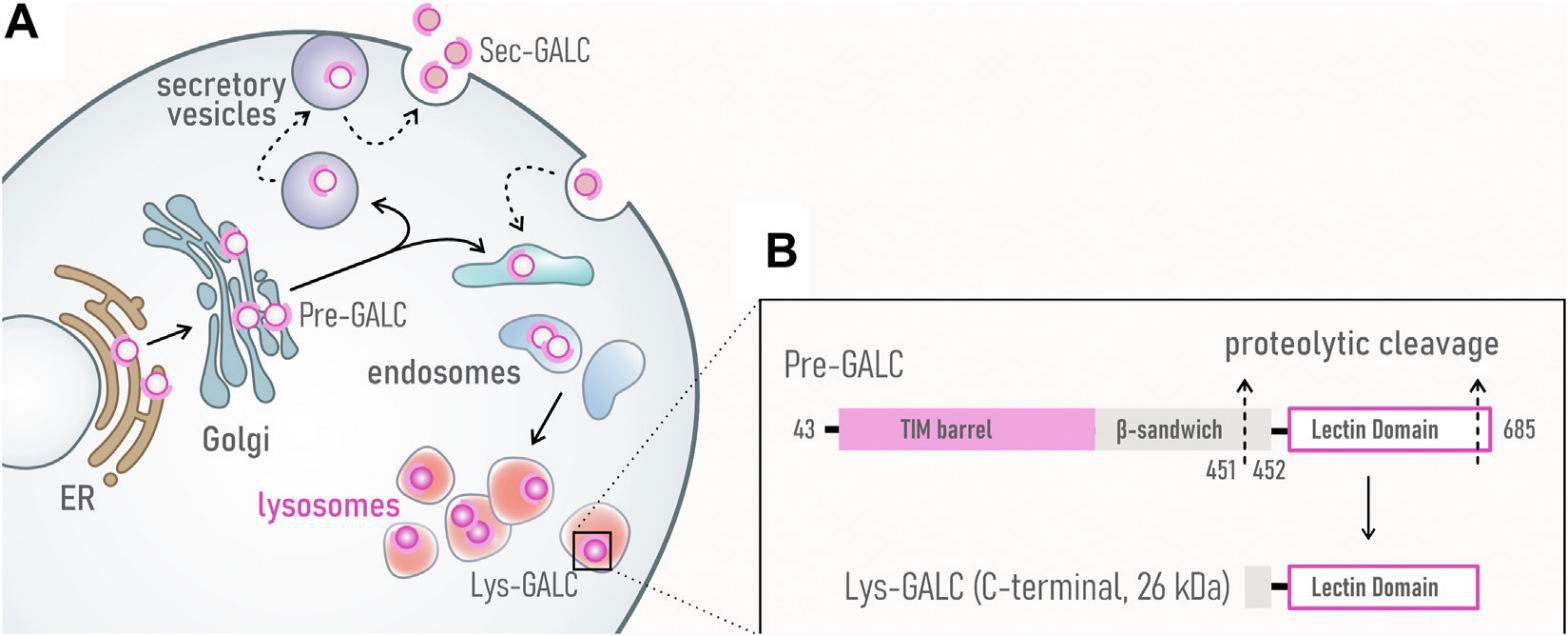
**Subcellular trafficking and lysosomal processing of GALC protein.***A*, schematic illustration of GALC trafficking through subcellular compartments. After protein synthesis in the ER, precursor GALC (Pre-GALC) is transported through the Golgi to 1) the extracellular space *via* secretory vesicles, where it forms secreted GALC (Sec-GALC), or 2) lysosomes *via* the endosomal pathway, where it becomes lysosomal GALC (Lys-GALC). *B*, lysosomal processing of GALC. Pre-GALC undergoes proteolytic cleavage at sites located in the β-sandwich and lectin domains to form mature GALC within lysosomes. A carboxyl terminal lys-GALC fragment can be detected by Western blot using the CL13.1 anti-GALC antibody. ER, endoplasmic reticulum.

GALC MMVs are evenly distributed among the three protein domains, the triosephosphate isomerase (TIM) barrel domain (57–353 aa), β-sandwich domain (354–468 aa), and lectin binding domain (488–684 aa) (Fig. 2) (23). It has been shown that p.T529M, p.L634S, and p.L645R induce GALC mis-trafficking and reduce lysosomal localization and secretion (24, 25, 26). Missense mutation at the p.R396 residue, which is essential for substrate binding (27), impairs GALC activity (28, 29), but not trafficking of the GALC mutant protein (26). These studies provide mechanistic insight into a few pathogenic MMVs; however, the question of how most other variants contribute to pathogenicity remains.

**Figure 2:**
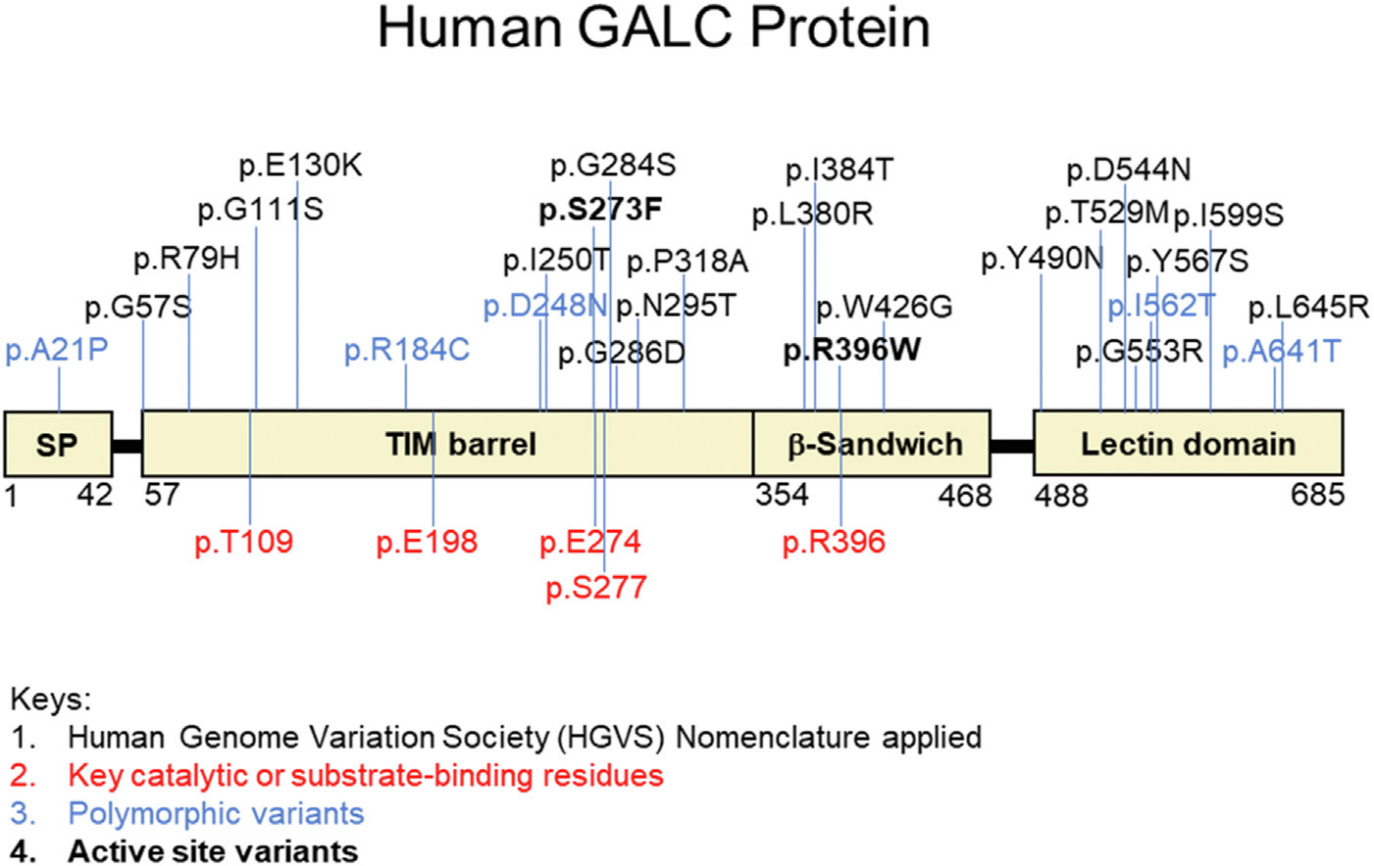
**Location and distribution of KD-related MMVs on the human GALC protein.** The schematic diagram shows the distribution of clinically relevant MMVs on the human GALC protein. Human Genome Variation Society (HGVS) nomenclature is applied. The main structural domains of GALC are indicated: signal peptide (SP) (1–42 a.a.), TIM barrel domain (57–353 a.a.), β-sandwich domain (354–468 a.a.), and lectin-binding domain (488–685 a.a). Key residues involved in catalytic function and substrate-binding are labeled in *red*. Polymorphic variants are labeled in *blue*. Active site variants are in *bolded font*. KD, Krabbe disease; MMV, missense mutation variant; TIM, triosephosphate isomerase.

In this study, we selected 21 pathogenic MMVs and characterized their effects on GALC activity and GALC trafficking. We also examined 10 *GALC* MMVs previously found to occur *in cis* with p.I562T, a known pseudodeficiency variant frequently identified as an allelic background in KD patients or infants at risk for KD (15, 30). Some MMVs, including p.S273F and p.R396W, likely alter GALC’s active site, disrupting its catalytic function (23). Two MMVs, p.N295T and p.D544N, previously shown to alter N-glycosylation status of GALC proteins (24, 26), were also included in the MMV panel. Altogether, we designed a systematic expression study of five polymorphic variants and 31 clinically relevant MMVs in a human oligodendrocytic cell line (MO3.13). The list of *GALC* MMVs examined in this study and related information is shown in Table 2. To maximize detection sensitivity in the cell-based assay, we eliminated the background signal from endogenous GALC protein using CRISPR-Cas9 genome editing, in order to generate a *GALC* KO cell line (MO3.13/*GALC*-KO). This strategy was crucial for testing MMVs associated with infantile-onset KD, where the resultant GALC activity could be undetectable above endogenous levels. After expression of the MMV panel in MO3.13/*GALC*-KO cells, we analyzed the quantitative profiles of residual GALC activity, lysosomal GALC protein levels, precursor GALC protein levels, psychosine and secreted GALC protein levels to understand the molecular effects of these MMVs on GALC function and trafficking, as well as to establish correlations with KD clinical severity.

**Table 2:** Clinically relevant GALC MMVs examined in this study

GALC variant	Polymorphic MM	*In cis w*/p.I562T	Patient geographic	Note	Reference
	p.A641T				
	p.A21P				
	p.R184C			*In cis* with 30 kb deletion	(20)
	p.D248N				
	p.I562T			Common pseudodeficiency variant	(30)
WT					
p.G57S		Yes	Italian		(12, 13, 42)
p.R79H		Yes	Italian		(15, 18)
p.G111S					(18)
p.E130K			Italian	Present in the Twi-5J mouse model	(15, 42, 61)
p.I250T			Greek		(16, 18, 28)
p.S273F			Japanese	Near active site	(16)
p.G284S		Yes			(12, 13, 16, 18, (66))
p.G286D		Yes	Japanese; European	Late-onset KD mutation	(13, 15, 16, 18, 28, (65))
p.N295T			Italian		(15)
p.P318A			Japanese		(16)
p.L380R			Japanese		(16)
p.I384T		Yes	Italian		(15)
p.R396W				Severely affect substrate binding	(11, 14, 15)
p.W426G		Yes			(17)
p.Y490N		Yes	Italian		(14, 15)
p.T529M		Yes	Northern European	High frequency	(11, 12, 13, 14)
p.D544N		Yes	Israeli Arab	Hyper-glycosylated	(24, 62, 63)
p.G553R			Italian		(15)
p.Y567S		Yes	Northern European	High frequency	(11, 12, 13, 14, 15)
p.I599S			Israeli Druze		(62)
p.L645R					(12, 64)

In cis w/p.I562T: MMV found in-cis with p.I562T in KD patients or infants at risk for KD.

## Results

### *GALC* KO background, human oligodendrocytic cell line for GALC MMVs expression study

**Figure 3:**
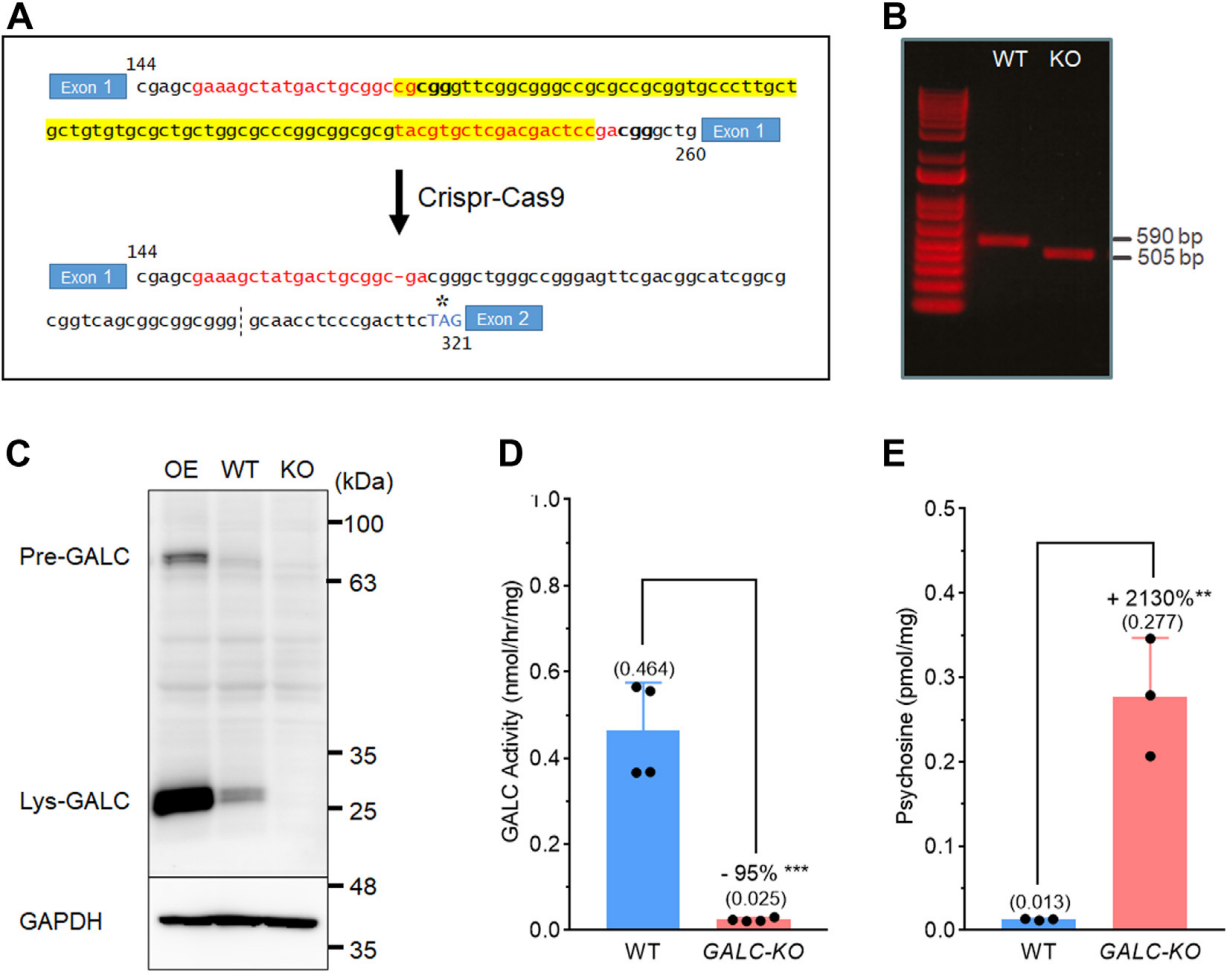
**Generation and validation of MO3.13/*GALC*-KO cells.***A*, human *GALC* gene sequences (*red*) adjacent to PAM sites (*bolded*) are targeted for Cas9-mediated cleavage by the sgRNA vectors. A downstream nonsense mutation (*asterisk*) is introduced upon successful targeted deletion (highlighted *yellow*). *B*, confirmation of targeted deletion of the 85 bp region in the KO cell line by PCR amplification, compared to control WT cells. *C*, Western blot analysis of GALC and GAPDH proteins in native MO3.13 cells (WT), GALC-KO cells (KO), and GALC-overexpressing cells (OE). *D*, GALC activity and (*E*) psychosine levels in WT and KO cells. Statistical significance is determined using an unpaired *t* test (two-tailed, 3–4 independent experimental replicates, 95% confidence interval, ∗∗*p* < 0.01, ∗∗∗*p* < 0.001). PAM, protospacer adjacent motif.

The demyelination observed in the central nervous system of KD is caused by functional deficiency of GALC in oligodendrocytes. MO3.13 cells express various oligodendrocyte markers, including 2′,3′-cyclic-nucleotide 3′-phosphodiesterase (CNPase), galactosylceramide (GalCer), and myelin basic protein (31, 32), and thus, are frequently used to study molecular mechanisms related to demyelination disorders, including multiple sclerosis (33, 34) and KD (35, 36). Likewise, for this study, we chose to model the molecular effect of GALC MMVs in this human oligodendrocytic cell line. To prevent endogenous WT GALC from interfering with the measurement of exogenous GALC variants, we knocked out *GALC* gene expression using the Crispr-Cas9 genome editing method. Targeted deletion of an 85 bp nucleotide in exon 1 was confirmed by PCR amplification of genomic DNA from the *GALC*-KO cell line (Fig 3, *A* and *B*). Absence of mature, lysosomal GALC protein (lys-GALC; 26 kDa) was confirmed by Western blot (WB), compared to native MO3.13 cells with and without the overexpression of GALC (Fig. 3*C*). For GALC activity assay of cell lysate, low but detectable levels of background signal were detected in the KO cells; however, native MO3.13 cells had 19-times higher activity (0.464 *versus* 0.025 nmol/mg/hr) (Fig. 3*D*). Psychosine levels were 21-times higher in KO cells compared to native cells (0.277 pmol/mg *versus* 0.013 pmol/mg, respectively), which highlights an anticipated deficiency of substrate catabolic activity (Fig. 3*E*). Our results confirm the *GALC* KO status of the MO3.13/*GALC*-KO cell line.

### Differential expression of lysosomal and ceramide pathway-related proteins in the MO3.13/GALC-KO cells

**Figure 4:**
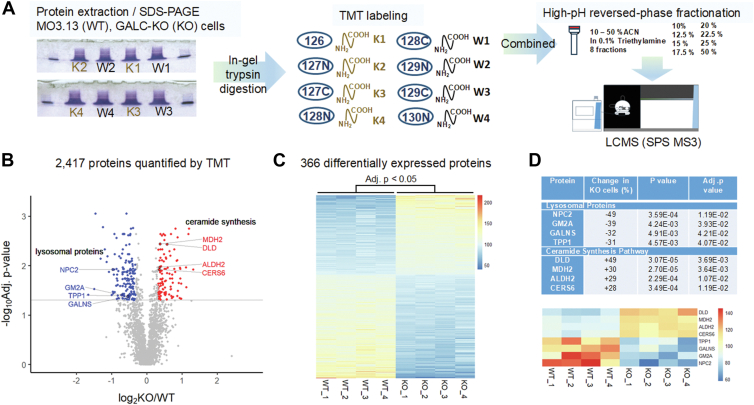
**Differential proteome of MO3.13 native cells (WT) and MO3.13/*GALC*-KO (KO) cells.***A*, proteomics workflow. Proteins extracted from WT and KO cells were separated by SDS-PAGE. After in-gel digestion, peptides were labeled with tandem mass tags (TMTs) and high-pH reversed-phase fractioned. Peptides were identified and quantified by liquid chromatography mass spectrometry (LC/MS) using synchronous precursor selection (SPS) and MS3 method, and the differential expression profile between WT and KO cells was elucidated. *B*, volcano plot. A total of 2417 proteins were identified and quantified. Three hundred sixty-six proteins were identified as differentially expressed in KO *versus* WT cells (two-tailed *t* test; adjusted *p* value (Benjamini–Hochberg) < 0.05). A *p* value of 0.05 (-log_10_ 0.05 = 1.301) is indicated by a *gray line*. Differentially expressed proteins with a 1.25-fold change (KO/WT: log2 < −0.32 or > 0.32) are highlighted (upregulated in *red*; downregulated in *blue*). *C*, differentially expressed proteins. Scaled abundances of the 366 differentially expressed proteins are represented in a heat map in descending order based on average log2-fold change. The color intensity scale is arbitrary. *D*, differently expressed lysosomal proteins and proteins in the ceramide synthesis pathway. Percent change (KO *versus* WT), *p* values, and adjusted *p* values are listed. Scaled abundances of the target proteins are shown in a heat map in descending order based on average log2-fold change. The color intensity scale is arbitrary.

A stable isotope-based quantitative proteomic approach, tandem mass tags (TMTs) and mass spectrometry, was used to compare the protein expression profiles of *GALC*-KO cells and native cells (Fig. 4*A*). A total of 4863 proteins were identified and 2417 quantified (Fig. 4*B*). Three hundred sixty-six proteins were differently expressed (Fig. 4*C*, Adj. *p* < 0.05), among these 106 were upregulated (KO/WT > 1.25; log2 > 0.32) and 161 downregulated (KO/WT < 0.8; log2 < −0.32). Interestingly, expression levels of several lysosomal proteins, NPC intracellular cholesterol transporter 2 (NPC2), ganglioside GM2 activator (GM2A), N-acetylgalactosamine-6-sulfatase (GALNS), and tripeptidyl-peptidase 1 (TPP1), were reduced in the GALC-KO cells (49%, 39%, 32%, and 31%, respectively). By contrast, levels of ceramide synthase 6 (CERS6) increased by 28% in *GALC*-KO cells. Other proteins in the *de novo* ceramide synthesis pathway, including dihydrolipoamide dehydrogenase (+49%), malate dehydrogenase (+30%) and aldehyde dehydrogenase 2 (+29%), were also elevated in the *GALC*-KO cells, which suggests there is a compensatory effect of ceramide production (Fig. 4*D*).

### Dramatic reduction of GALC activity in 20 clinically relevant *GALC* MMVs

**Figure 5:**
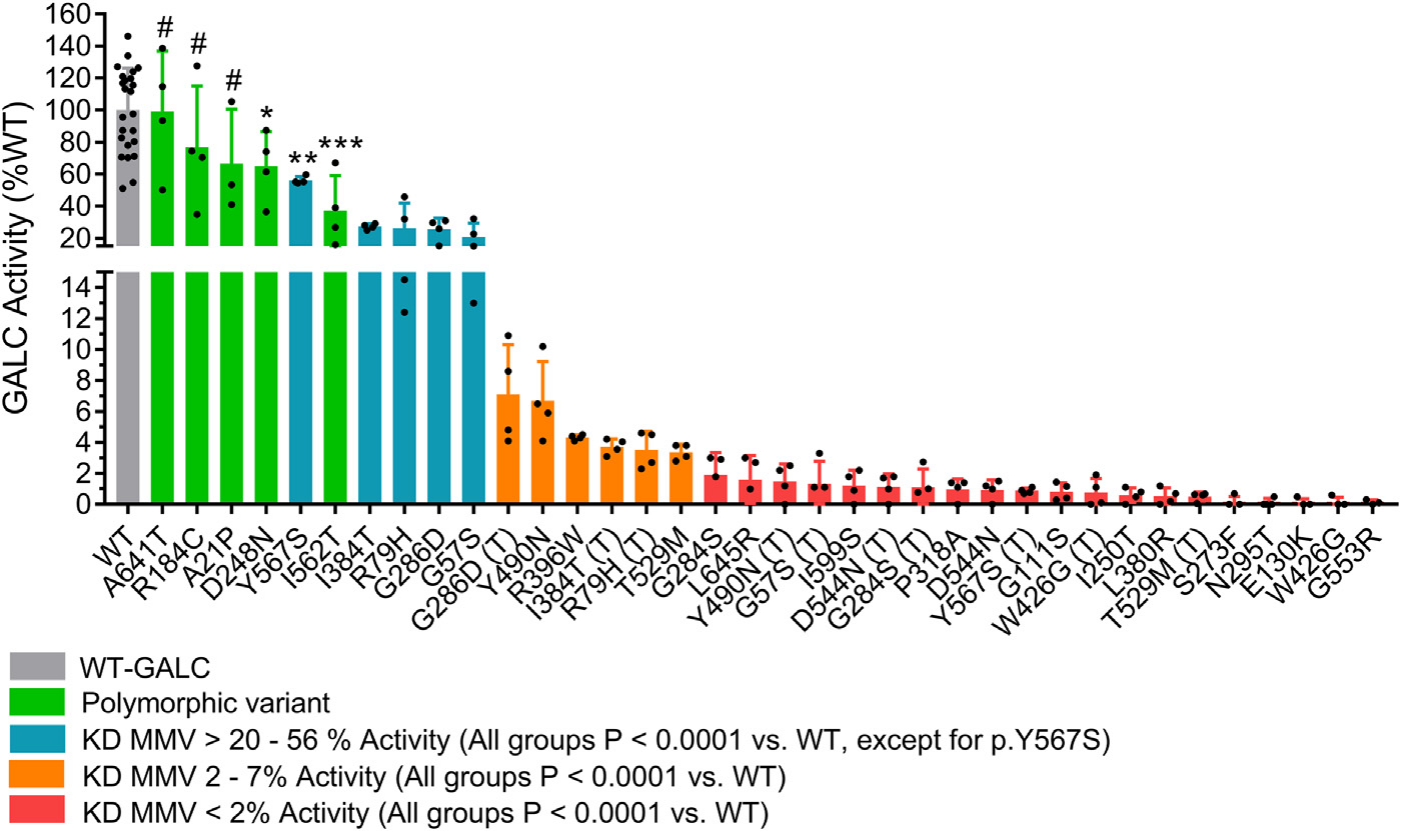
**Dramatic reduction of GALC activity in 26 clinically relevant MMVs.** Residual GALC activity in transiently expressed cell models was measured in cell lysate and presented as relative percent of WT-GALC. MMVs are ranked in descending order based on GALC activity. Of the MMVs eliciting significant reductions compared to WT-GALC, seven variants had activity that remained relatively high (20–70% of WT, *blue bars* and I562T); six variants had low level activity (2–7% of WT, *orange bars*); and 20 variants had little to no activity (0–2% of WT, *red bars*). MMVs on the p.I562T background are annotated with (T). Data are presented as mean ± standard deviation from four independent experiments. Individual MMV (n = 4) was compared to WT (n = 24) by an unpaired *t* test (two-tailed, 95% confidence interval, ∗*p* < 0.05, ∗∗*p* < 0.01, ∗∗∗*p* < 0.001, # - no significant difference). MMV, missense mutation variant.

Low *in vitro* GALC activity in leukocytes (≤0.15 nmol/mg/hr) is commonly used as the first confirmatory test for KD in clinical settings (37, 38). Therefore, we also measured residual GALC activity in transfected cell lysate as a readout to determine the molecular effect of MMVs on GALC function in MO3.13/*GALC*-KO cells. GALC activity of the WT transfectant was two-fold greater than native MO3.13 cells (0.92 nmol/mg/hr *versus* 0.46 nmol/mg/hr, respectively) (Table 3, GALC Activity). Three out of five polymorphic variants, p.A641T, p.A21P, and p.R184C, yielded similar activity to WT-GALC transfected cells; while the remaining two variants, p.D248N and p.I562T, caused GALC activity to be reduced by 32% and 61%, respectively (Table 3; Fig. 5, green bars). By contrast, 20 of the 31 MMV transfectants reduced GALC activity by more than 98%, including p.G284S, p.L645R, p.Y490N(T) (p.I562T cis-background abbreviated as “(T)”), p.G57S(T), p.I599S, p.D544N(T), p.G284S(T), p.P318A, p.D544N, p.Y567S(T), p.G111S, p.W426G(T), p.I250T, p.L380R, p.T529M(T), p.S273F, p.N295T, p.E130K, p.W426G, and p.G553R. Most of the MMVs known to be associated with the infantile-onset form of KD, such as p.G553R, p.T528M(T), and p.G567S(T), were found in this group (Fig. 5, red bars). MMVs that elicited activity between 2%-7% of WT activity included variants associated with later-onset form of KD, such as p.G286D(T), p.I384T(T), and p.R79H(T) (Fig. 5, orange bar). MMV transfectants with activity between 20% and 65% are polymorphic variants, p.I562T and p.D248N, and other single variants, including p.Y567S, p.G286D, p.I384T, p.R79H, and p.G57S, which are all likely to be nonpathogenic in the absence of the p.I562T background (Fig. 5, blue bar). Our data suggest that residual GALC activity from MMVs expressed in MO3.13/*GALC*-KO cells has the potential to be used as a readout to predict, based on percent activity, a clinical KD form.

**Table 3:** Molecular characterization of GALC MMVs in MO3.13/*GALC*-KO transiently expressed cell models

GALC variant	GALC activity			*p* Value (versus)		Lys-GALC	Sec-GALC		*p* Value (versus)		Pre-GALC		*p* Value (versus)	
	nmol/mg/hr	% WT	SEM	WT	p.I562T	% WT	% WT	SEM	WT	p.I562T	% WT	SEM	WT	p.I562T
WT	0.92	100.0	5.4			100.0	100.0	4.4			100.0	6.1		
Polymorphic variants														
p.A641T	0.92	99.2	18.8	ns		94.3	86.9	14.3	ns		94.3	22.0	ns	
p.A21P	0.61	66.5	19.7	ns		106.2	88.9	17.1	ns		66.9	20.1	∗	
p.R184C	0.71	76.8	19.1	ns		76.2	79.3	8.7	∗		153.5	11.5	∗∗	
p.D248N	0.60	64.8	10.8	∗		36.1	97.7	14.1	ns		122.9	4.8	ns	
p.I562T	0.34	37.2	11.0	∗∗∗		27.4	42.3	3.7	∗∗∗∗		101.9	7.6	ns	
Single MMV														
p.G111S	0.01	0.8	0.3	∗∗∗∗		0.0	120.1	20.3	ns		193.6	61.1	∗∗∗∗	
p.E130K	0.00	0.1	0.1	∗∗∗∗		4.5	94.5	10.8	ns		54.1	30.6	∗∗	
p.I250T	0.01	0.6	0.2	∗∗∗∗		10.9	94.1	17.8	ns		194.3	69.7	∗∗∗	
p.S273F	0.00	0.1	0.2	∗∗∗∗		0.0	89.4	4.6	ns		92.4	43.9	ns	
p.N295T	0.00	0.1	0.1	∗∗∗∗		0.0	12.2	6.1	∗∗∗∗		609.6	180.6	∗∗∗∗	
p.P318A	0.01	1.0	0.3	∗∗∗∗		12.4	78.7	10.0	∗		82.2	28.7	ns	
p.L380R	0.00	0.5	0.3	∗∗∗∗		0.0	0.9	0.9	∗∗∗∗		135.7	13.4	∗	
p.R396W	0.04	4.3	0.1	∗∗∗∗		47.4	27.2	1.9	∗∗∗∗		43.5	1.4	∗∗∗	
p.G553R	0.00	0.0	0.2	∗∗∗∗		0.0	0.0	0.0	∗∗∗∗		96.2	19.1	ns	
p.I599S	0.01	1.2	0.5	∗∗∗∗		0.0	0.0	0.0	∗∗∗∗		108.9	31.5	ns	
p.L645R	0.01	1.6	0.8	∗∗∗∗		0.0	0.0	0.0	∗∗∗∗		85.4	25.8	ns	
MMV on p.I562T background														
p.G57S	0.19	20.8	4.4	∗∗∗∗		19.2	35.9	6.9	∗∗∗∗		199.4	38.2	∗∗∗∗	
p.G57S (T)	0.01	1.3	0.7	∗∗∗∗	∗∗	0.0	0.0	0.0	∗∗∗∗	∗∗	69.4	17.2	ns	∗
p.R79H	0.24	26.2	7.9	∗∗∗∗		23.1	55.4	14.6	∗∗∗		112.7	42.0	ns	
p.R79H (T)	0.03	3.5	0.6	∗∗∗∗	∗	0.0	0.0	0.0	∗∗∗∗	∗∗	68.8	7.6	ns	ns
p.G284S	0.02	1.9	0.7	∗∗∗∗		13.3	57.1	6.4	∗∗∗		79.6	20.1	ns	
p.G284S (T)	0.01	1.1	0.6	∗∗∗∗	ns	2.4	28.3	8.4	∗∗∗∗	ns	90.4	12.4	ns	ns
p.G286D	0.24	25.4	3.5	∗∗∗∗		52.8	87.1	6.4	ns		159.9	42.0	∗∗	
p.G286D (T)	0.07	7.1	1.6	∗∗∗∗	∗∗	16.0	28.4	6.5	∗∗∗∗	∗∗	94.9	15.3	ns	ns
p.I384T	0.25	27.2	0.9	∗∗∗∗		11.5	18.5	1.1	∗∗∗∗		211.0	10.4	∗∗∗∗	
p.I384T (T)	0.03	3.7	0.3	∗∗∗∗	∗∗∗∗	0.0	2.0	0.4	∗∗∗∗	∗∗∗	101.5	3.4	ns	∗∗∗∗
p.W426G	0.00	0.1	0.2	∗∗∗∗		1.4	0.0	0.0	∗∗∗∗		140.8	43.9	∗	
p.W426G (T)	0.01	0.8	0.4	∗∗∗∗	ns	1.1	0.0	0.0	∗∗∗∗	ns	52.2	27.7	∗∗	ns
p.Y490N	0.06	6.7	1.3	∗∗∗∗		8.1	29.5	4.7	∗∗∗∗		166.9	41.1	∗∗∗	
p.Y490N (T)	0.01	1.5	0.6	∗∗∗∗	∗	1.1	3.5	2.0	∗∗∗∗	∗∗	73.2	18.2	ns	∗
p.T529M	0.03	3.4	0.3	∗∗∗∗		1.7	2.8	0.3	∗∗∗∗		125.7	4.0	ns	
p.T529M (T)	0.00	0.5	0.2	∗∗∗∗	∗∗∗∗	0.0	0.0	0.0	∗∗∗∗	∗∗∗	33.3	0.8	∗∗∗∗	∗∗∗∗
p.D544N	0.01	0.9	0.3	∗∗∗∗		3.5	79.7	8.0	ns		82.8	44.9	ns	
p.D544N (T)	0.01	1.1	0.4	∗∗∗∗	ns	0.3	61.4	6.0	∗∗	ns	83.4	22.9	ns	ns
p.Y567S	0.52	56.1	1.2	∗∗		45.4	56.1	2.3	∗∗∗		104.6	3.0	ns	
p.Y567S (T)	0.01	0.9	0.1	∗∗∗∗	∗∗∗∗	0.0	0.0	0.0	∗∗∗∗	∗∗∗∗	21.8	0.8	∗∗∗∗	∗∗∗∗

GALC activity in MMV cell models positively correlates with age of symptom onset in KD patients carrying compound heterozygous or homozygous MMV genotypes.

**Figure 6:**
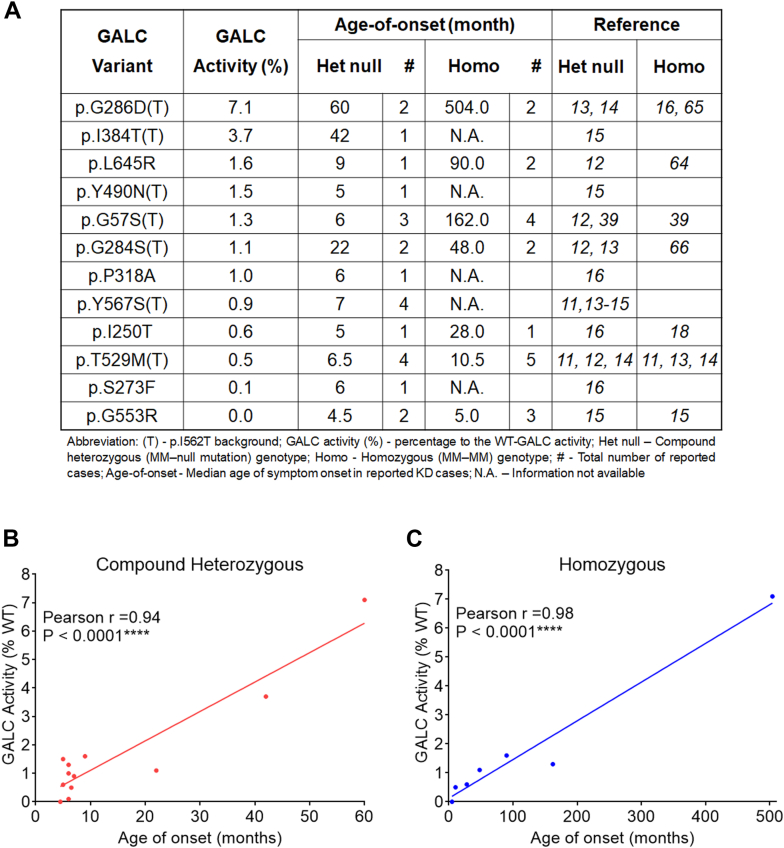
**GALC activity in MMV cell models is highly correlated with the age of clinical onset in KD patients carrying the same GALC MMV genotypes.***A*, GALC activity levels in MMV cell models and the age of symptom onset in KD patients with either compound heterozygous MM-null mutation or homozygous MM-MM genotypes, based on reported cases from literature. *B* and *C*, correlation analysis between GALC activity and the age of symptom onset in KD patients with (*B*) heterozygous MM-null mutation genotypes (Pearson r = 0.94, *p* < 0.0001, n = 12) or (*C*) homozygous MM-MM genotypes (Pearson r = 0.98, *p* < 0.0001, n = 7). KD, Krabbe disease; MMV, missense mutation variant.

GALC MMVs are inherited as compound heterozygous or homozygous genotypes in KD patients. Here, we compare the age of symptom onset for published KD cases that are compound heterozygous with an MM-null mutation, against the GALC activity observed for these same variants in the MO3.13/*GALC*-KO cells. Patients with these genotypes have a pathogenic MMV on one allele and a null mutation, such as the 30 kb deletion, on the second allele (Fig. 6*A*, “Het null”). MMVs that are associated with compound heterozygous genotypes include p.G553R, p.Y490N(T), p.I250T, p.S273F, p.P318A, p.G57S(T), p.T529M(T), p.Y567S(T), p.L645R, p.G248S(T), p.I384T(T), and p.G286D(T), shown in ascending age-of-onset order. A total of 23 KD cases containing these heterozygous genotypes have been reported in published studies alongside age-of-onset information (Table 1). Remarkably, the GALC activity associated with these variants was highly correlated with the clinical onset observed in KD patients (Fig. 6*B*, Pearson r = 0.94, *p* < 0.0001). For example, the most severe mutation, p.G553R, had undetectable GALC activity in the model and an average age-of-onset at 4.5 months. The least severe mutation, p.G286D(T), which maintained 7.1% of WT GALC activity in the cell model, has an average age-of-onset at 60 months (juvenile-onset). In fact, all MMVs associated with infantile-onset KD (age-of-onset ≤ 12 months) resulted in an activity less than 2% of WT in the model. By contrast, another variant known to cause juvenile-onset disease (p.I384T(T), age-of-onset = 42 months) produced GALC activity at 3.7% (Fig. 6*A*).

After excluding cases that underwent treatment intervention, only 19 KD cases with seven homozygous MM-MM genotypes have been reported with age-of-onset information. Homozygous GALC MMVs associated with KD include p.G553R, p.T529M(T), p.I250T, p.G284S(T), p.L645R, p.G57S(T), and p.G286D(T), shown in ascending age-of-onset order (Fig. 6*A*, “Homo”). Clinical onset is consistently delayed (*i.e.* milder clinical severity) in patients with homozygous genotypes compared to patients with compound heterozygous genotypes of the same MMV, in part, due to higher residual GALC activity. For example, patients with homozygous p.I250T and p.L645R experience an average age-of-onset at 28 months and 90 months, respectively. By contrast, patients with the same MMVs but inherited in compound heterozygous state present with symptoms at 5 months and 9 months, respectively. p.G553R and p.G286D(T), inherited in the homozygous state, had the lowest and the highest activity in the cell model (0% versus 7.1% of WT, respectively); which also translated into the earliest (5 months) and latest (504 months) age of symptom onset (Fig. 6*A*). Strikingly, the activity associated with these variants was almost perfectly correlated with the age of symptom onset in KD patients (Fig. 6*C*, Pearson r = 0.98, *p* < 0.00001). An apparent outlier was the homozygous p.G57S(T) genotype, which has been previously reported as a variant associated with longer survival (39). Overall, we detect a strong genotype–phenotype correlation when comparing GALC activity in our MMV cell models and age of disease onset in KD patients with the *GALC* genotypes examined in our study. These results suggest that clinical onset in patients carrying these MMV genotypes may be predicted from residual GALC activity observed in the MO3.13/*GALC*-KO cell model and associated assays reported in this study.

### Lys-GALC protein levels correlate with GALC activity

**Figure 7:**
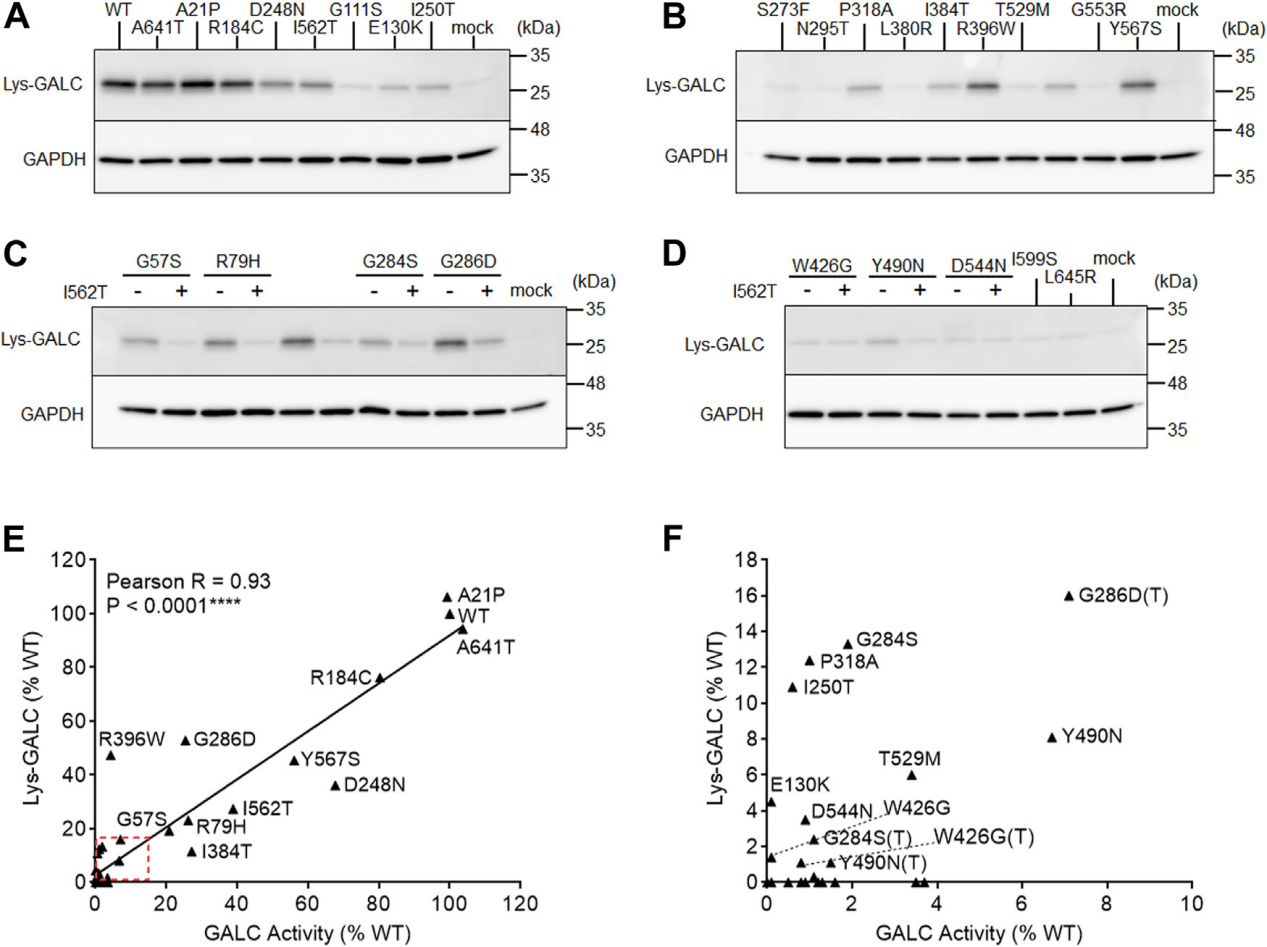
**Lys-GALC levels are highly correlated with GALC activity.***A*–*D*, Western blot results of lys-GALC and GAPDH in the lysate of GALC MMV expressing cells. Panel (*C* and *D*) shows MMVs in the absence (−) and presence (+) of the p.I562T polymorphic background. *E*, Lys-GALC levels (normalized to GAPDH levels) are highly correlated with GALC activity in the GALC MMV cell models (Pearson r = 0.93, *p* < 0.0001, n = 37). *F*, expanded plot of the *red line* area shown in (*E*). MMVs with lys-GALC levels greater than 1% are labeled in (*E*) and (*F*). MMV, missense mutation variant.

Herein, we also analyzed the levels of lys-GALC protein in cells expressing the GALC MMVs panel. The cleaved, mature GALC fragment of WT-GALC can be detected as a 26 kDa lys-GALC protein band by the CL13.1 antibody, which targets the C-terminal fragment of GALC (Fig. 7, *A*–*D*). The p.D248N and p.I562T polymorphic variants reduced lys-GALC levels by 64% and 73%, respectively, compared to WT; whereas the pathogenic p.E130K and p.I250T variants reduced lys-GALC levels by 95% and 89%, respectively (Fig. 7*A*, Table 3). Cells expressing the p.I562T covariant tended to lower lys-GALC levels compared with the corresponding single MMV (Fig. 7, *C* and *D*, and Table 3). Importantly, lys-GALC levels were positively and highly correlated to GALC activity among WT and all 36 MMVs examined (Fig. 7, *E* and *F*, Pearson r = 0.93, *p* < 0.0001). The strong correlation between activity and lys-GALC levels indicates that cleaved, mature GALC is the primary contributor to its activity. GALC MMVs that produced less than 2% of WT activity, including p.G57S(T), p.R79H(T), p.G111S, p.S273F, p.N295T, p.L380R, p.I384T(T), p.T529M(T), p.G553R, p.Y567S(T), p.I599S, and p.L645R, were largely undetectable by WB (Table 3). These results further support the notion that the deficiencies to GALC activity caused by GALC MMVs are primarily due to reductions in lys-GALC levels, except in the case of catalytic variants such as p.R396W, which are likely to act through both lys-GALC reduction and impaired catalytic activity.

### KD-related MMVs variably reduce secretion of GALC

**Figure 8:**
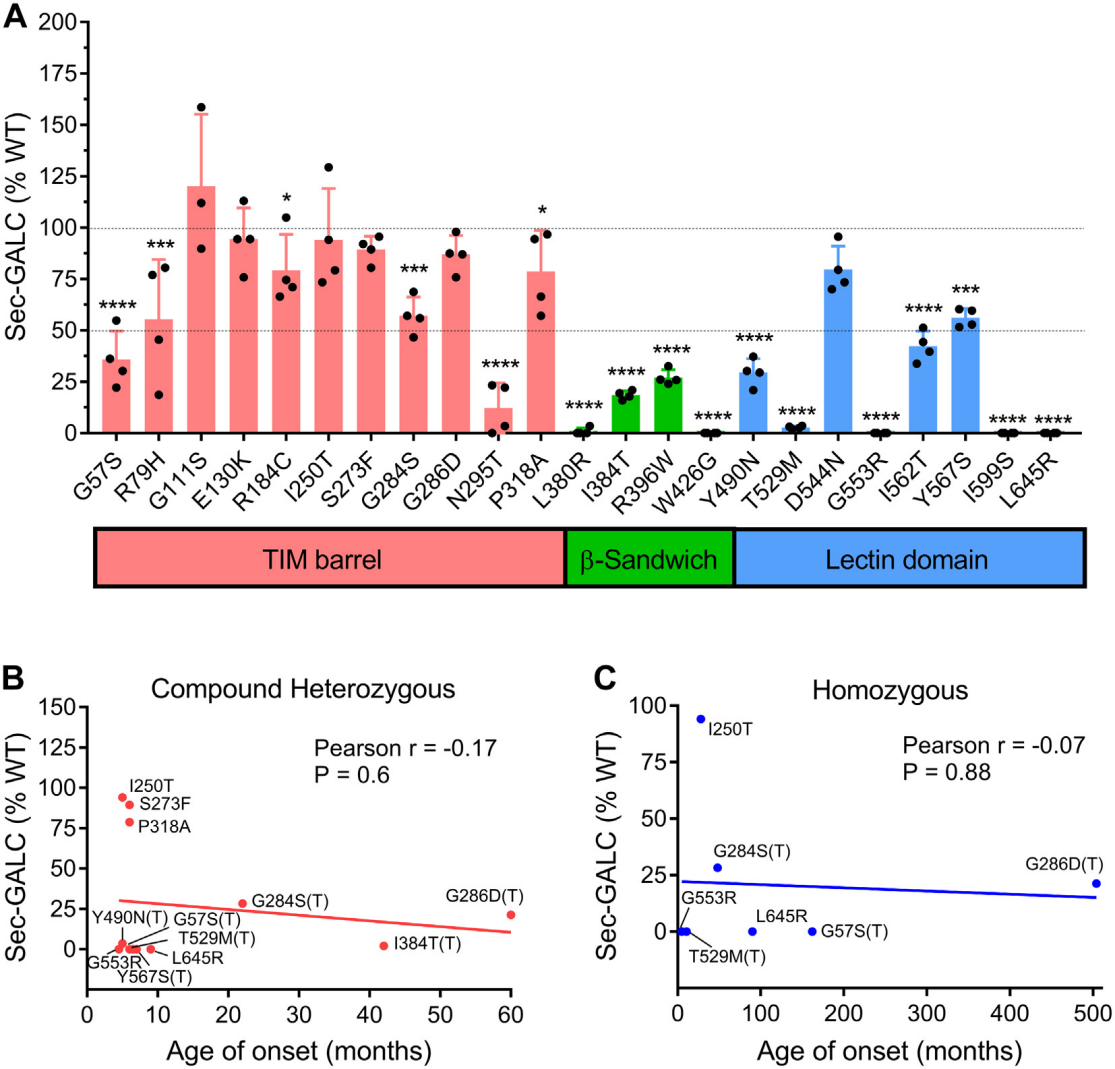
**GALC secretion is more impaired by MMVs located on the β-sandwich and lectin protein domains but does not correlate to the age of symptom onset in KD patients.***A*, Sec-GALC levels are plotted against the structural location of GALC MMVs, categorized by their position on the TIM barrel (*red*), β-sandwich (*green*), and lectin-binding (*blue*) domains. MMVs (11/12) significantly reduced sec-GALC levels compared with WT-GALC, specifically on the β-sandwich and lectin domains in our cell models. (Unpaired *t* test, two-tailed, four independent experimental replicates, 95% confidence interval; ∗*p* < 0.05, ∗∗*p* < 0.01, ∗∗∗*p* < 0.001, ∗∗∗∗*p* < 0.0001). *B* and *C*, analysis reveals no significant correlation between sec-GALC levels and the age of symptom onset in KD patients with (*B*) heterozygous MM-null mutation genotypes or (*C*) homozygous MM-MM genotypes. KD, Krabbe disease; MMV, missense mutation variant; TIM, triosephosphate isomerase.

In order to assess changes to protein trafficking elicited by KD-related MMVs, we quantified sec-GALC levels using an adaptive in-house sandwich ELISA. The carboxyl terminal end of GALC is trimmed in lysosome during the maturation process, therefore, the sandwich ELISA captures the V5 tag on the C-terminal end of sec-GALC, followed by detection with a highly specific antibody against an internal epitope on GALC (CL13.1). In the cell model, sec-GALC was measured at 8.6 ng/ml after WT-GALC was expressed for 72 h. Polymorphic variants, p.R184C and p.I562T, caused a reduction to sec-GALC levels by 21% and 58%, respectively (Table 3). Eighteen of the 26 single MMVs (including the p.I562T covariants) also led to lower sec-GALC levels (21% to 100%). Notably, six MMVs with mutations located on the β-sandwich domain or lectin domain of GALC (p.L380R, p.W426G, p.T529M, p.G553R, p.I599S, and p.L645R), as well as the 7 p.I562T covariants, including p.G57S(T), p.R79H(T), p.I384T(T), p.W426G(T), p.Y490N(T), p.T529M(T), and p.Y567S(T), reduced sec-GALC in the cells by more than 95% (Fig. 8*A* and Table 3). Combined with our data that show these 13 MMVs ameliorated GALC activity and lowered lys-GALC to undetectable levels, our results suggest that the observed GALC deficiency in these MMVs is likely *via* a mis-trafficking mechanism. The reduction of sec-GALC caused by individual MMVs coincides to the structural domain that contains the mutation. For example, five of ten single MMVs located on the TIM barrel domain (57–353 aa) had no significant effect on sec-GALC levels. Alternatively, 9 of 11 single MMVs located on a carboxyl domain (354–685aa) reduced sec-GALC by at least 70% (Fig. 8*A*). p.G111S, p.E130K, p.I250T, p.S273F, p.G286D, and p.P318A, which are all found on the TIM barrel domain and in relatively close proximity to the active site (23) (Fig. 2), selectively reduced GALC activity and lys-GALC levels (Table 3). Taken together, our data suggest that these MMVs might selectively disrupt GALC trafficking to lysosome, but not to the extracellular space. Significant correlations between sec-GALC levels and age of symptom onset were not detected (Fig. 8, *B* and *C*). Therefore, we conclude that reduced GALC secretion is not a major factor contributing to clinical onset in KD.

### KD-related MMVs variably induce accumulation of intracellular pre-GALC

**Figure 9:**
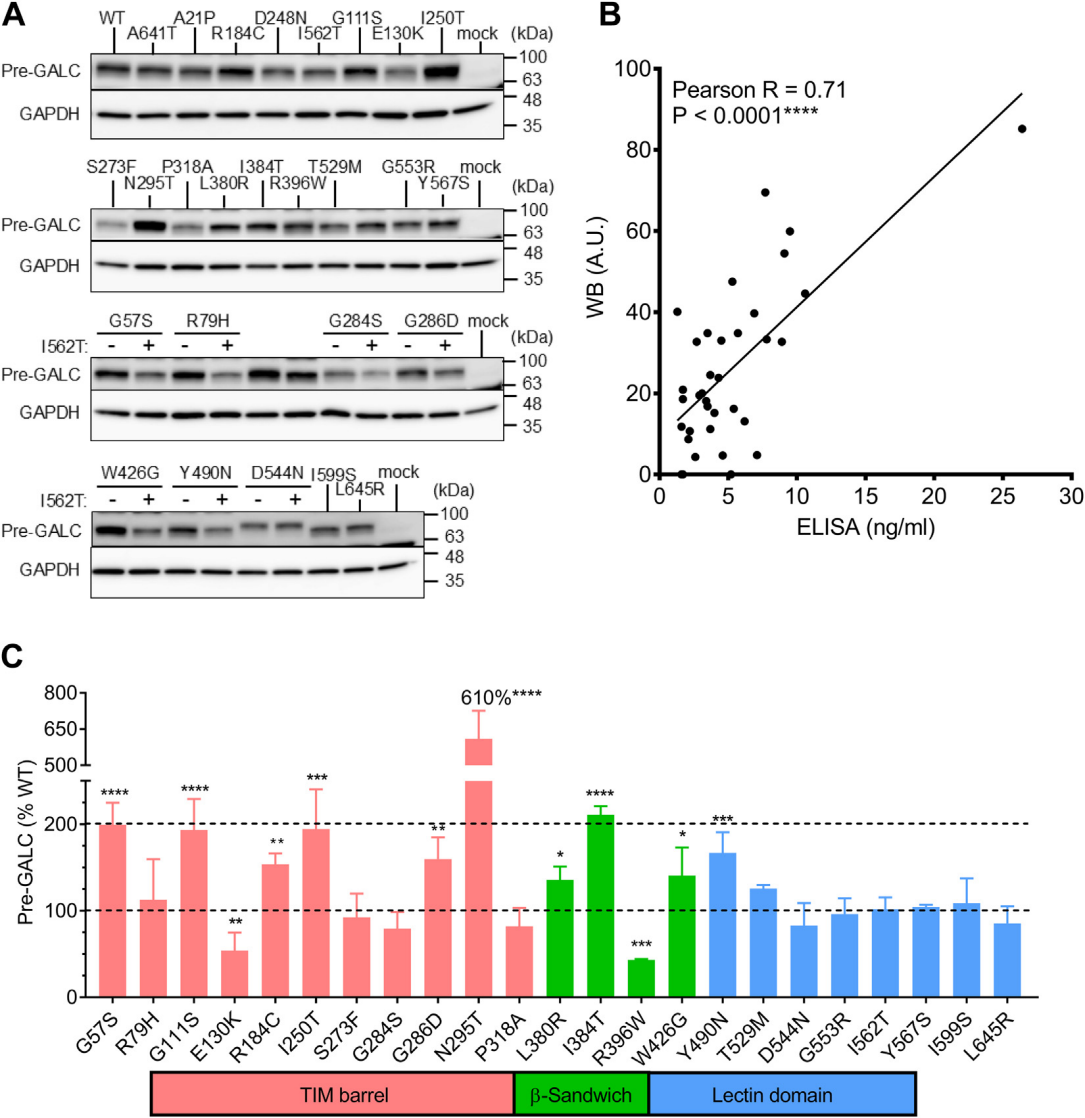
**Accumulation of pre-GALC protein in GALC MMV cell models.***A*, intracellular pre-GALC and GAPDH proteins were detected by Western blot. The bottom two blots show MMVs in the absence (−) and presence (+) of the p.I562T polymorphic background. The same GAPDH blots were used to normalize both pre-GALC (*current panel*) and lys-GALC protein levels (Fig. 7, *A*–*D*), as pre-GALC and lys-GALC were detected on the same blot but at different molecular weights. *B*, pre-GALC levels (normalized to GAPDH) detected by Western blot are significantly correlated with pre-GALC levels measured by sandwich ELISA in the MMV cell models (Pearson r = 0.71, *p* < 0.0001, n = 35). *C*, pre-GALC levels measured by ELISA plotted against the structural location of the MMVs, categorized by their position on the TIM barrel (*red*), β-sandwich (*green*) and lectin-binding (*blue*) domains. Ten out of 22 MMVs significantly increase pre-GALC levels compared with WT-GALC in the MMV cell models. (Unpaired *t* test, two-tailed, three independent experimental replicates, 95% confidence interval; ∗∗*p* < 0.01, ∗∗∗*p* < 0.001, ∗∗∗∗*p* < 0.0001). MMV, missense mutation variant; TIM, triosephosphate isomerase.

Intracellular retention of MM carrying proteins is a common pathogenic phenotype in many lysosomal storage disorders, due in part, to the failure of endoplasmic reticulum (ER)-associated degradation, which normally eliminates misfolded proteins over time (40, 41). We measured retained, intracellular pre-GALC levels in transfected cells by ELISA using detergent-soluble cell lysate. The pre-GALC ELISA was validated by comparison to WB, which, as expected, revealed that the pre-GALC ELISA signal was positively correlated to the density of the 75 kDa pre-GALC protein band (Fig. 9, *A* and *B*, Pearson r = 0.71, *p* < 0.0001, n = 35). Pre-GALC levels were elevated in 10 MMVs and reduced in 2 MMVs, when expressed individually (Fig. 9*C*). Expression of one variant, p.N295T, which has previously been shown to induce abnormal glycosylation in cells (26), caused a 6-fold increase in pre-GALC levels. Other MMVs (5) that caused elevated pre-GALC levels were also associated with a reduction in sec-GALC, including p.R184C (+54% pre-GALC; −21% sec-GALC), p.G57S (+99%, −64%), p.N295T (+510%, −88%), p.I384T (+89%, −70%) and p.Y490N (+67%, −78%). These data suggest that intracellular retention of GALC reduced secretion in these MMVs expressing cells (Table 3). Conversely, we identified a group of MMVs (p.L380R, p.W426G, p.T529M, p.G553R, p.I599S, and p.L645R) that also reduced levels of sec-GALC, but did not increase pre-GALC levels; thus, the secretion defect in these variants is independent of intracellular retention.

### The p.I562T background synergistically impairs GALC function to reach pathogenic level

**Figure 10:**
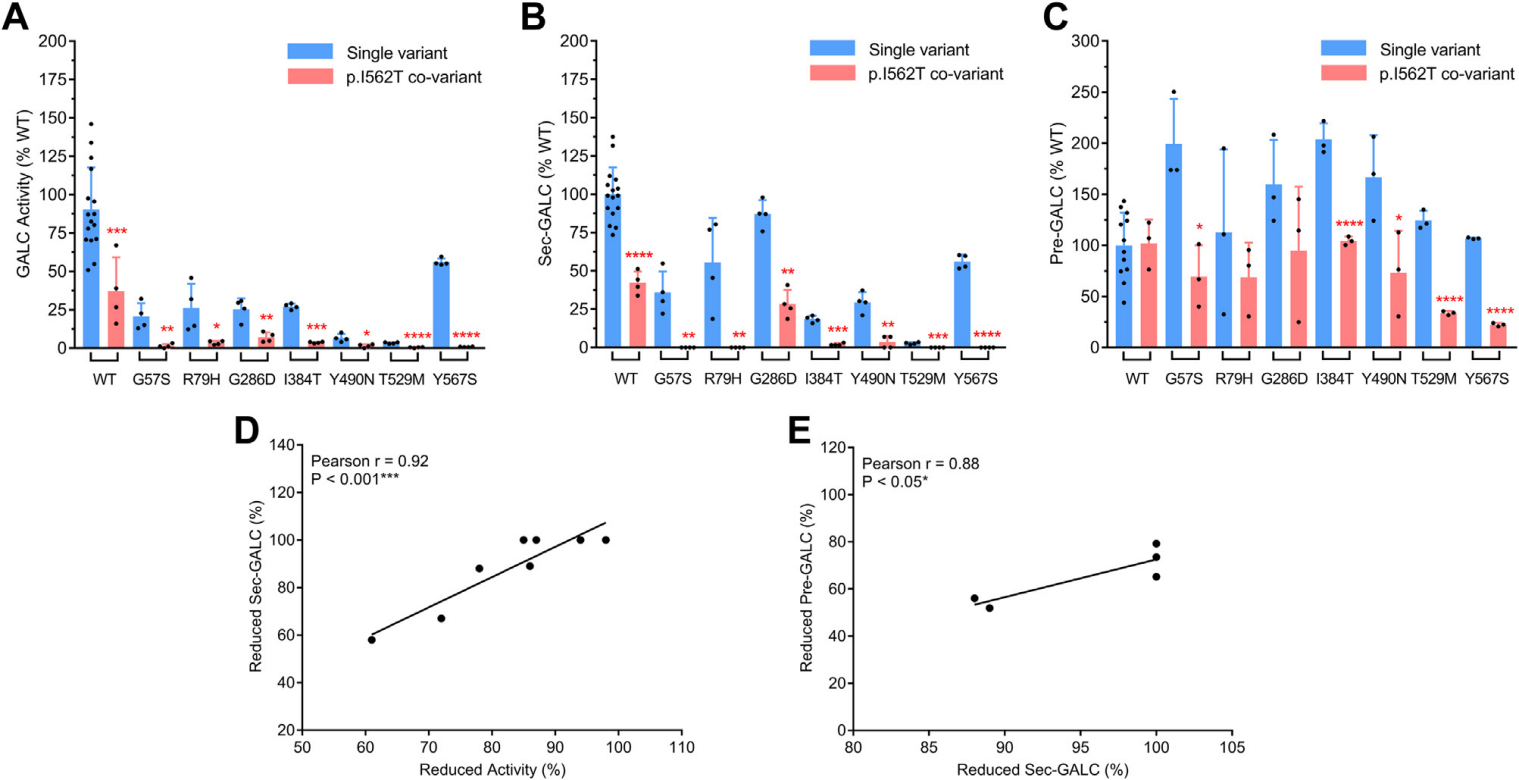
**p.I562T background reduced GALC activity, sec-GALC levels and pre-GALC levels in the MMV cell models.***A*, GALC activity, (*B*) sec-GALC levels and (*C*) pre-GALC levels from MMV expressing cells containing the p.I562T variant and seven other p.I562T co-variants (*red bars*) are compared side-by-side with those from WT cells or cells expressing the corresponding single variants (*blue bars*), respectively. (Unpaired *t* test, two tails, three to four independent experimental replicates, 95% confidence interval; ∗∗*p* < 0.01, ∗∗∗*p* < 0.001, ∗∗∗∗*p* < 0.0001). *D*, the percentage reductions of GALC activity are highly correlated with the percentage reductions in sec-GALC levels in MMV expressing cells containing the p.I562T and seven other p.I562T co-variants (Pearson r = 0.92, *p* < 0.001, n = 8). *E*, reductions in sec-GALC levels (%) correlated with the reductions in intracellular pre-GALC levels (%) in five p.I562T covariants (Pearson r = 0.88, *p* < 0.05, n = 5). MMV, missense mutation variant.

p.I562T is a known pseudodeficiency variant associated with reduced levels of GALC activity. In fact, it was present in 87% of the 348 referral samples in the New York State Newborn Screen Program, after screening about two million samples (30). Not only is it identified as a pseudodeficiency variant, it is also frequently identified as an allelic background with other MMVs on the *GALC* gene in confirmed KD cases (15, 42). To evaluate the molecular effect of the p.I562T background on GALC protein, we analyzed the expression of a series of GALC p.I562T covariants, including the p.G57S(T), p.R79H(T), p.G284S(T), p.G286D(T), p.I384T(T), p.W426G(T), p.Y490N(T), p.T529M(T), p.D544N(T), and p.Y567S(T) (Table 3, Fig. 10). Expression of p.I562T alone greatly reduced WT-GALC activity by nearly 60% in the MO3.13/*GALC*-KO cells. Correspondingly, p.I562T coexpression synergistically reduced GALC activity in many variants (6 of 10); specifically, p.G57S (20.8% to 1.3%), p.R79H (26.2% to 3.5%), p.G286D (25.4% to 7.1%), p.I384T (27.2% to 3.7%), p.Y490N (6.7% to 1.5%), and p.Y567S (56.1% to 0.9%) (Fig. 10*A*). In contrast, p.I562T coexpression did not lower the activity observed with the p.G284S, p.W426G, p.T529M, and p.D544N variants, which were already at very low activity (≤3%) when expressed alone (Table 3). These results suggest that the presence of the p.I562T allelic background can impair GALC function enough to reach the threshold of disease onset for these six MMVs.

Interestingly, the p.I562T covariants that resulted in reduced GALC activity, also caused a significant reduction in GALC secretion (Fig. 10*B*), and the two were highly correlated (Fig. 10*D*, Pearson r = 0.92, *p* < 0.001, n = 8).These data suggest that the p.I562T background proportionally reduces the levels of correctly folded GALC that traffics to both extracellular space and lysosomes. Mechanistically, p.I562T likely destabilizes the protein structure and induces the formation of misfolded GALC, which ultimately leads to an increase in degradation. Indeed, intracellular pre-GALC levels were lower among most p.I562T covariants compared to the corresponding single variants, although not all reached statistical significance (Fig. 10*C*). Among covariants that statistically lowered pre-GALC levels in the cell model (5 of 7), pre-GALC levels correlated to the reduced sec-GALC levels (Pearson r = 0.88, *p* < 0.05, n = 5); suggesting that p.I562T impairs GALC function through increased degradation of pre-GALC (Fig. 10*E*).

### GALC activity correlates with GALC secretion in the MMV cell models

**Figure 11:**
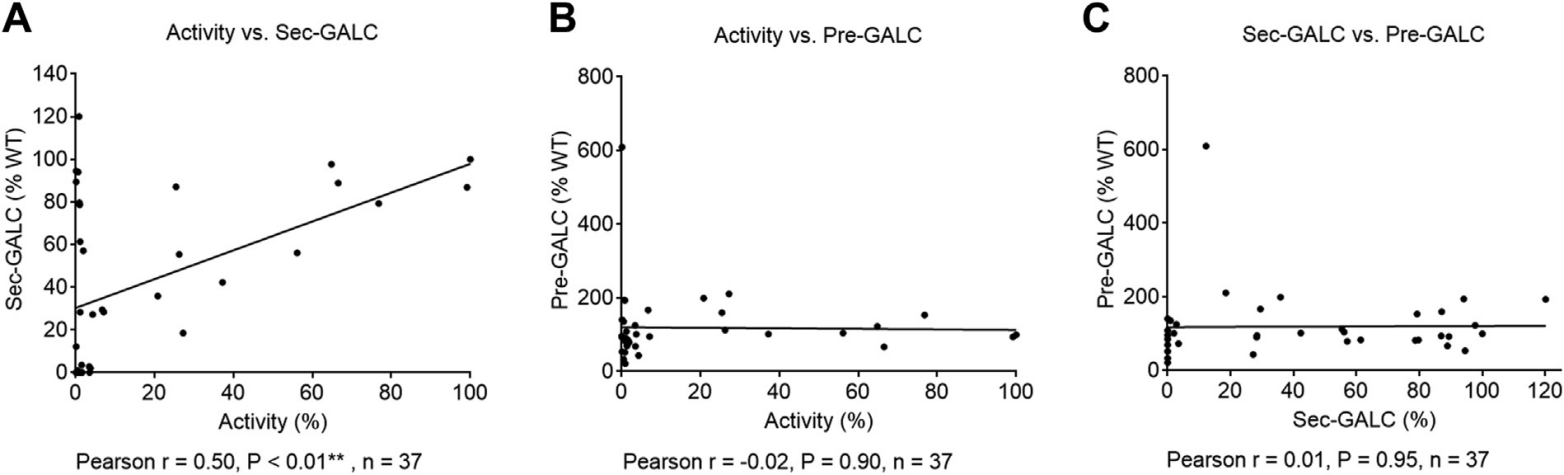
**GALC activity correlates with sec-GALC levels in the MMV cell models.** Correlations among GALC activity, sec-GALC levels, and pre-GALC protein levels in the GALC MMV expressing cells were analyzed using the Pearson correlation method. *A*, GALC activity is significantly correlated with sec-GALC levels in the cell models (Pearson r = 0.5, *p* < 0.01, n = 37). No significant correlations are found between (*B*) GALC activity and pre-GALC levels, and between (*C*) sec-GALC and pre-GALC levels. MMV, missense mutation variant.

Overall, most MMVs we analyzed led to a reduction in GALC activity > 93% (26 of 36). Of these MMVs, half also lowered sec-GALC > 95%, and thus are likely to be associated with GALC insecretion—a protein mis-trafficking phenotype. Indeed, there was a positive correlation between GALC activity and sec-GALC level elicited by all MMVs (Fig. 11*A*, Pearson r = 0.5, *p* < 0.01, n = 37). The moderate correlation was largely driven by single MMVs located on the TIM barrel domain, which resulted in a unique low GALC activity/normal sec-GALC profile. On the other hand, we did not detect any correlations between intracellular pre-GALC level and GALC activity (Fig. 11*B*, Pearson r = −0.02, n = 37), or between pre-GALC and sec-GALC levels (Fig. 11*C*, Pearson r = 0.01, n = 37). Taken together, these data suggest that among these variants, intracellular retention or degradation of pre-GALC is not a major determinant of the amount of GALC being trafficked to lysosomes or into the extracellular space.

### Residual GALC activity correlates with endogenous psychosine levels in the MMV cell models

Psychosine is a *bona fide* natural substrate of GALC, and psychosine levels reliably reflect the *in situ* activity of GALC. Moreover, quantitative psychosine analysis was recently used to differentiate samples from KD patients with different clinical onset (43). It is now well-accepted in the newborn screen setting that 10 nmol/L of psychosine detected in a dried blood spot is the cutoff value for infantile-onset KD cases that require referral for treatment intervention (44, 45). Cell models of psychosine accumulation have been lacking, due to a general low abundance of endogenous psychosine in most cell types. To our knowledge, the MO3.13/*GALC*-KO cell line used in this study is one of the few human cell models with a psychosine accumulation phenotype. We report a 21-fold increase in psychosine production in the *GALC*-KO cell line compared to parent cells (Fig. 3*E*), which resembles the change in psychosine observed in brain tissue (5). This robust readout cell model gives us the opportunity to examine the effect of pathogenic MMVs on psychosine accumulation in human cells.

**Figure 12:**
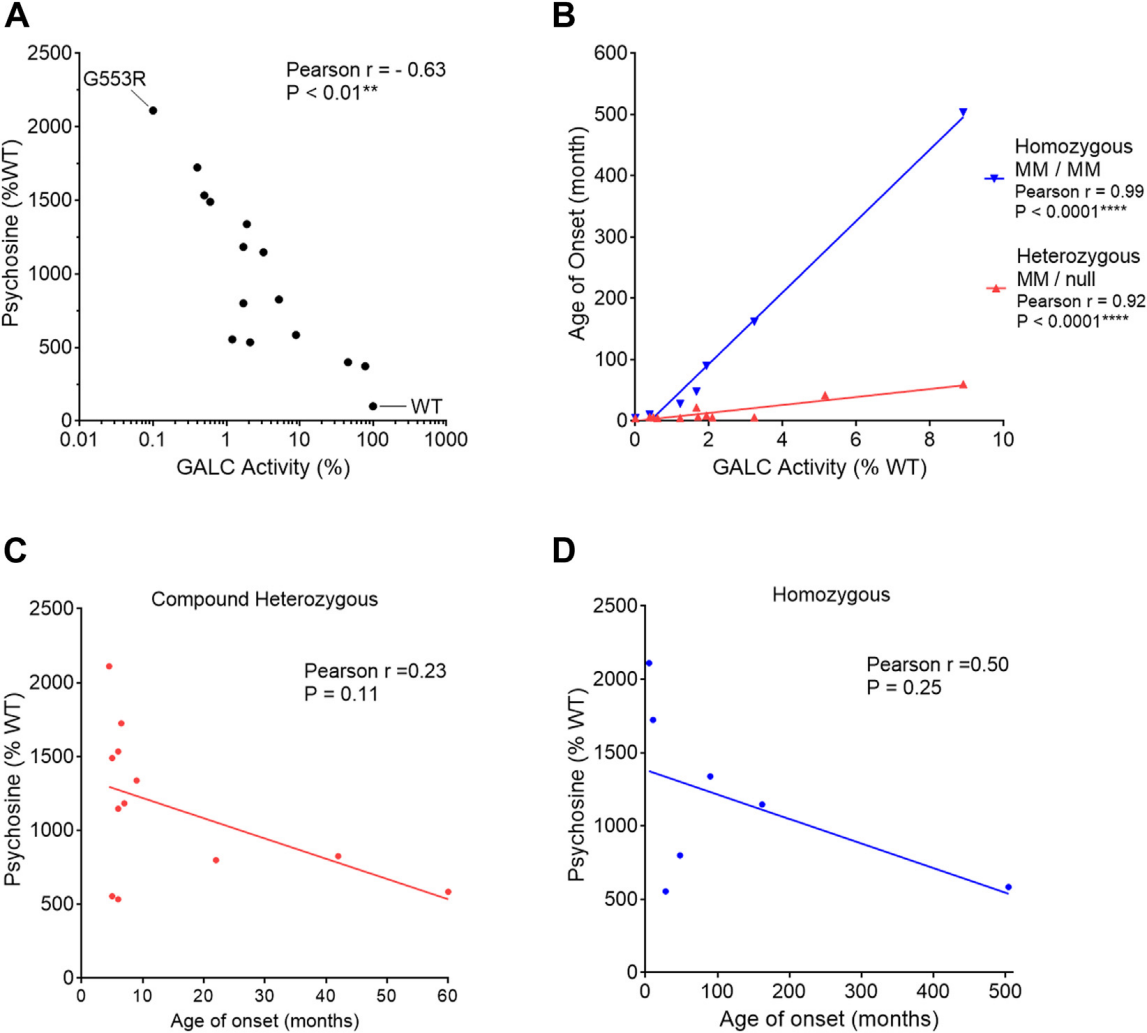
**Cellular psychosine levels correlate with GALC activity in MMV cell models, but do not correlate with the onset of clinical symptoms in KD patients.***A*, expression studies were conducted in MO3.13/*GALC*-KO cells for WT, p.I562T, and 12 clinically relevant GALC MMVs. Quantification of intracellular psychosine, GALC activity, and sec-GALC levels are summarized in Table 4. Psychosine levels significantly correlated with GALC activity (Pearson r = −0.63, *p* < 0.01, n = 15). *B*, consistent with our transient expression study (Table 3, Fig. 5), GALC activity was also highly correlated with the age of symptom-onset in KD patients with either homozygous MM-MM (Pearson r = 0.99, *p* < 0.0001, n = 7) or compound heterozygous MM-null mutation genotypes (Pearson r = 0.92, *p* < 0.0001, n = 12). *C* and *D*, however, psychosine levels were not significantly correlated with the age of symptom-onset, in either (*C*) compound heterozygous or (*D*) homozygous MMV genotypes. KD, Krabbe disease; MMV, missense mutation variant.

To understand the correlation between clinical forms of KD and psychosine levels in the cell model, we performed a separate expression study on a subset of MMVs with published data for age of symptom onset. Empty vector (mock), WT-GALC, p.I562T, and 12 pathogenic MMVs were transfected into cells for 72 h. Transfected cells and medium were harvested and analyzed for cellular psychosine, residual GALC activity, and sec-GALC (Table 4). At baseline, psychosine levels were high due to the absence of GALC. Transient transfection of WT-GALC led to the expression of intracellular GALC in a subpopulation of cells. However, GALC secreted from positively transfected cells was also taken up by neighboring nontransfected cells through a mechanism known as cross-correction. Therefore, psychosine was cleared by exogenous GALC regardless of transfection status of the cells. Transfection of WT-GALC reduced psychosine levels by 95% compared to mock control (from 0.348 to 0.016 pmol/mg). Notably, the difference in cellular psychosine levels (21-fold) found between *GALC-KO* and parent MO3.13 cells was identical to that observed between WT-GALC and mock transfected cells (Fig. 3*E*), which suggests that the expression of WT-GALC normalized psychosine levels to match native cells. Transfection of the pseudodeficiency variant p.I562T (46% GALC activity) and the late-onset variant p.G286D(T) (9% GALC activity), reduced psychosine levels by 82% and 73%, respectively, relative to mock control. Expression of two MMVs associated with infantile-onset KD examined in this panel, p.T529M(T) (0.39% GALC activity) and p.G553R (0% GALC activity), only reduced psychosine levels by 20% and 2%, respectively. Affirmatively, there was a negative correlation between residual GALC activity and psychosine level among mock control, WT-GALC, p.I562T, and the 12 pathogenic MMVs we analyzed (Fig. 12*A*, Pearson r = −0.63, *p* < 0.01, n = 15). These results confirm that GALC function impaired by pathogenic MMVs increases psychosine accumulation in the current cell-based assay.

**Table 4:** Psychosine level, GALC activity and sec-GALC level in MO3.13/GALC-KO cells expressing a subset of clinically relevant MMVs

GALC variant	Psychosine			*p* value (versus)		GALC activity			*p* value (versus)		Sec-GALC			*p* value (versus)		Age-of-onset (month)	
	pmol/mg	SEM	% WT	WT	p.I562T	nmol/mg/hr	SEM	% WT	WT	p.I562T	ng/ml	SEM	% WT	WT	p.I562T	Het-null	Homo
Mock	0.35	0.01	2154			0.00	0.00	0.00			0.00	0.00	0.00				
WT	0.02	0.00	100		∗∗∗∗	5.45	0.60	100.00		∗∗	20.21	0.51	100.00		∗∗∗		
p.I562T	0.06	0.00	399	∗∗∗∗		2.49	0.06	45.59	∗∗		11.69	0.58	57.85	∗∗∗			
p.G57S (T)	0.19	0.01	1148	∗∗∗∗	∗∗∗∗	0.18	0.01	3.23	∗∗∗	∗∗∗∗	1.82	0.05	9.02	∗∗∗∗	∗∗∗∗	6	162
p.I250T	0.09	0.01	555	∗∗		0.07	0.01	1.22	∗∗∗		55.02	1.33	272.29	∗∗∗∗		5	28
p.S273F	0.25	0.02	1535	∗∗∗		0.03	0.01	0.49	∗∗∗		38.49	0.64	190.50	∗∗∗∗		6	N.A.
p.G284S (T)	0.13	0.01	800	∗∗∗∗	∗∗∗	0.09	0.01	1.66	∗∗∗	∗∗∗∗	9.52	0.17	47.11	∗∗∗∗	∗	22	48
p.G286D (T)	0.09	0.01	585	∗∗	∗	0.49	0.03	8.90	∗∗	∗∗∗∗	10.55	0.51	52.19	∗∗∗	N.S.	60	504
p.P318A	0.09	0.01	535	∗∗∗		0.11	0.02	2.09	∗∗∗		40.81	0.99	201.95	∗∗∗∗		6	N.A.
p.I384T (T)	0.13	0.01	827	∗∗	∗∗	0.28	0.01	5.15	∗∗∗	∗∗∗∗	0.76	0.10	3.74	∗∗∗∗	∗∗∗∗	42	N.A.
p.Y490N (T)	0.24	0.02	1491	∗∗∗	∗∗∗	0.03	0.01	0.60	∗∗∗	∗∗∗∗	1.56	0.12	7.74	∗∗∗∗	∗∗∗∗	5	N.A.
p.T529M (T)	0.28	0.01	1725	∗∗∗∗	∗∗∗∗	0.02	0.00	0.39	∗∗∗	∗∗∗∗	0.00	0.00	0.00	∗∗∗∗	∗∗∗∗	6.5	10.5
p.G553R	0.34	0.01	2112	∗∗∗∗		0.00	0.01	0.00	∗∗∗		0.00	0.00	0.00	∗∗∗∗		4.5	5
p.Y567S (T)	0.25	0.00	1184	∗∗∗	∗∗∗	0.09	0.02	1.70	∗∗∗	∗∗∗∗	0.00	0.00	0.00	∗∗∗∗	∗∗∗∗	7	N.A.
p.L645R	0.19	0.39	1339	∗∗∗∗		0.11	0.00	1.93	∗∗∗		0.00	0.00	0.00	∗∗∗∗		9	90

Consistent with our results above (Fig. 6), the residual GALC activity observed in this model was highly correlated to the age of disease onset for both compound heterozygous and homozygous genotypes (Fig. 12*B*, Pearson r = 0.92–0.99, *p* < 0.0001). These results suggest that our expression study, under our preset conditions, is consistent and reproducible. Therefore, we performed correlation analysis between psychosine levels (relative to WT-GALC levels) and age of disease onset for compound heterozygous or homozygous MMV genotypes. In many cases, psychosine levels in cells expressing MMVs associated with infantile-onset KD, *e.g.* p.G553R and p.T529M(T), trended higher compared with that associated with late-onset KD (p.G286D(T)) (Table 4). Unfortunately, a significant correlation with either genotype was not observed (Fig. 12, *C* and *D*), indicating that psychosine levels in the cell models do not coincide with age of symptom onset in KD.

## Discussion

In this study, we investigated the functional and trafficking effects of 21 pathogenic GALC MMVs and 10 additional MMVs found in cis with the pseudodeficiency variant p.I562T in a *GALC*-KO human oligodendrocytic cell line. Our findings provide new insights into the molecular mechanisms that underly KD and highlight the potential use for residual GALC activity measurements to inform patient diagnostics and prognostics.

### Impact of GALC MMVs on enzyme activity and clinical severity

Our results show that most GALC MMVs (20/31) dramatically reduce residual GALC activity, resulting in a ≥ 98% reduction in activity compared to WT-GALC. These results are consistent with the observation that clinically relevant mutations in the *GALC* gene result in severe enzyme deficiencies in KD patients. Notably, MMVs found in infantile-onset patients with homozygous and compound heterozygous MM-null mutation genotypes, including p.S273F (16), p.N295T (15), p.P318A (16), p.Y490N (T) (15), p.G553R (15), p.T529M (T) (12, 15), and p.Y567S (T) (12, 15), yielded < 2% of WT GALC activity in the model, which aligns with the early and severe clinical presentation seen in these patients. Conversely, cells expressing MMVs associated with later-onset forms of KD, such as p.G286D (T) (13, 15, 16) and p.I384T (T) (15), showed GALC activities between 2% and 7%, which justifies that observed in milder forms of the disease.

The strong correlation observed between residual GALC activity and age of disease onset in patients with compound heterozygous and homozygous MMV genotypes reinforces the notion that GALC activity obtained from this type of expression study in *GALC*-KO cells can serve as a reference of disease severity and as a potential tool for predicting the phenotype of a variant of uncertain significance (Figs. 6 and 12*B*).

### Mechanisms of GALC dysfunction: trafficking and maturation

**Figure 13:**
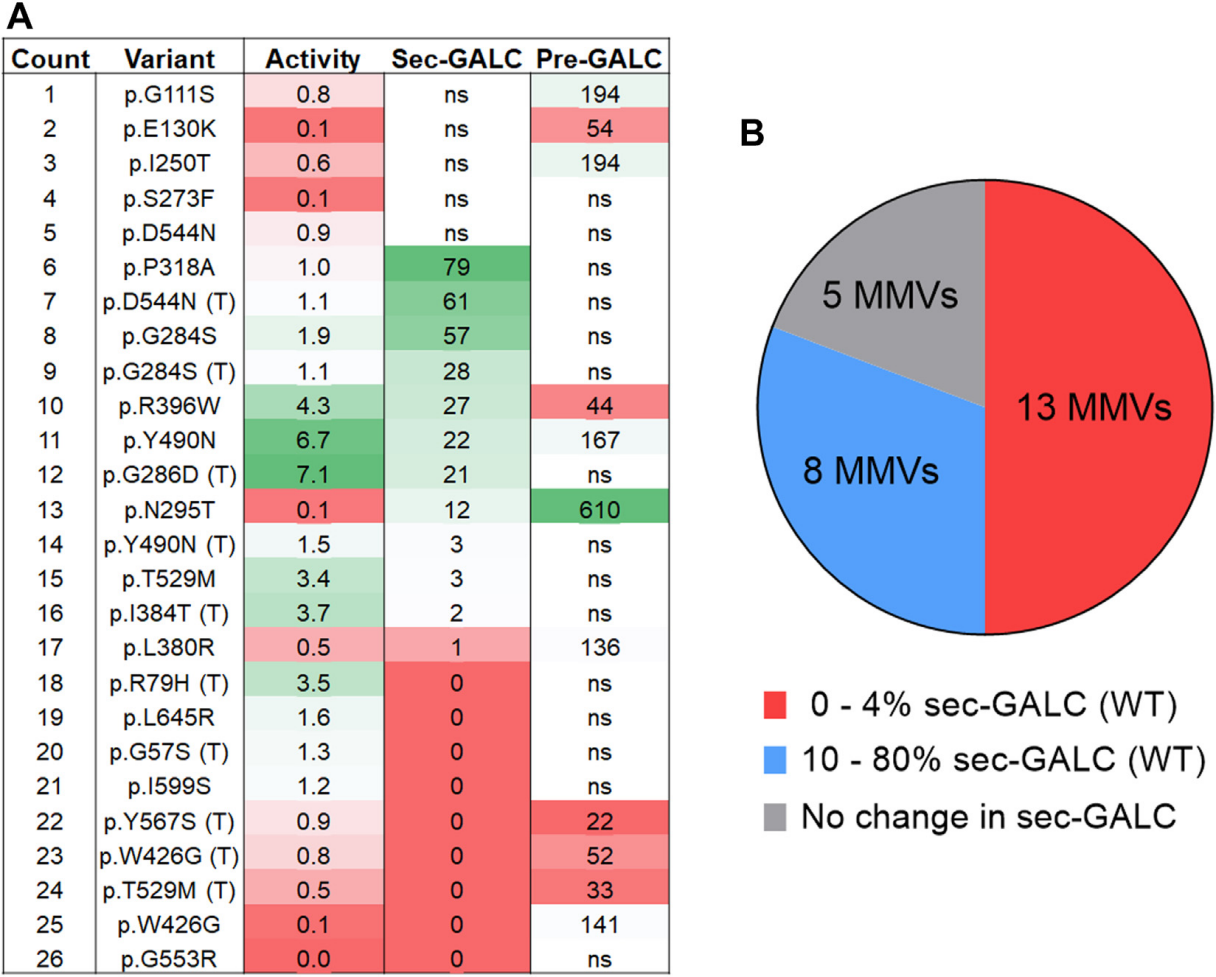
**Dramatic reduction in GALC secretion in 50% of the low-activity GALC MMVs.***A*, summary table listing GALC activity, sec-GALC levels, and intracellular pre-GALC levels for 26 low-activity GALC MMVs. Values are presented as percentages relative to WT-GALC levels. The color spectrum from *green* to *red* indicates values from high to low within each group. “ns” denotes no significant difference from WT-GALC. *B*, distribution of sec-GALC levels among the 26 low-activity GALC-MMVs. The *red*, *blue*, and *gray* sections of the pie chart represent MMVs with sec-GALC levels of 0% to 4%, 10% to 80%, and 100% of WT levels, respectively. MMV, missense mutation variant.

Our study also sheds light on the mechanisms by which GALC MMVs affect enzyme function. We observed that many MMVs reduce levels of mature GALC (lys-GALC) in lysosomes, with a strong positive correlation between lys-GALC levels and GALC activity. This suggests that impaired trafficking of GALC to the lysosome is a key factor contributing to reduced enzyme activity. In addition, we observed that many MMVs (13) lead to dramatic reductions in both GALC secretion and GALC activity by more than 96% (Fig. 13*A*, #14–26). Thus, these variants likely provoke misfolding and instability that affects protein trafficking to both extracellular space and lysosomes. In fact, several MMVs that cause severe protein misfolding based on protein structure prediction (23), including p.L380R (Fig. 13, #17), p.W426G (Fig. 13*A*, #25), p.G553R (Fig. 13*A*, #26), and p.L645R (Fig. 13*A*, #19), were all associated with ultra-low GALC secretion (reduced > 99%), lys-GALC levels and activity (both reduced > 98%) in our study, highlighting the robustness of the assay to quantify functional and trafficking deficits of MMVs.

Interestingly, 10 out of 36 MMVs also elevated levels of intracellular pre-GALC in the model, which suggests that these variants cause retention of the enzyme in the ER and/or other intracellular compartments. The accumulation of pre-GALC could contribute to the observed deficiencies in enzyme secretion and activity. However, the fact that not all MMVs with reduced GALC activity caused an increase in pre-GALC levels, indicates that multiple mechanisms are likely at play, such as potential differences in protein stability and the trafficking pathway. p.G111S, p.E130K, p.I250T, and p.P318A caused a dramatic reduction in GALC activity but not in secretion (Fig. 13*A*, #1–3), which points to an alternative mechanism that selectively reduces lys-GALC function, such as catalytic inactivation. Of relevance, an *ARSA* variant, p.P426L, a pathogenic MMV in metachromatic leukodystrophy, has been shown to selectively destabilize arylsulfatase A protein in lysosomes due to impairment of oligomerization (46). It remains to be investigated whether the reduction to lys-GALC observed with these variants is related to a similar mechanism.

### Modifier effects of the p.I562T pseudodeficiency variant

The presence of the p.I562T pseudodeficiency variant significantly impacted the function of coexpressed GALC MMVs, leading to a synergistic impairment of GALC activity and secretion (Fig. 10, *A* and *B*). Given that many of these covariants also elicited reductions in intracellular pre-GALC levels (Fig. 10*C*), we conclude that the p.I562T variant exacerbates the effects of other MMVs through a protein destabilization or expression inhibitory mechanism. A previous study on physical properties of the p.I562T protein indicated that the polymorphism had only a subtle negative effect on its enzymatic activity and thermal stability compared to WT GALC. However, it had more impact on protein trafficking, because the variant could not be detected in the culture media of overexpressed HEK293T cells (26). These findings are consistent with our results (50% reduction) (Tables 3 and 4). Pseudodeficiency variants were also identified in other lysosomal storage disorders, such as p.R247W and p.R249W in the *HEXA* gene (47), p.A300T in the *IDUA* gene (48), p.R595W in the *GLB1* gene (49), p.D152N in the *GUSB* gene (50), and p.D313Y in the *GLA* gene (51). In addition, it has been reported that p.D313Y synergistically reduced α-galactosidase A function when expressed in cis with p.R112C or p.C172G, which were both identified in patients with classical Fabry disease (52).

Given the high frequency with which p.I562T occurs in KD, cases of suspected KD sent for molecular testing should also undergo allelic DNA sequencing to check for p.I562T background status. Our results also highlight the need to include p.I562T background information in published literatures and genetic disorder archives when reporting a variant as pathogenic or likely pathogenic. For example, both p.G286D and p.Y567S are classified as pathogenic or likely pathogenic on ClinVar. Although GALC activity on both variants was below-threshold levels when expressed in cis with p.I562T (pG286D (T) at 7%, p.Y567S (T) at 1%), p.G286D and p.Y567S variants, when expressed alone, had GALC activity of 87% and 56% in our study, respectively (Table 3). Other studies consistently report high GALC activity for these two variants (25, 29); and both variants were uniformly found on the p.I562T background in all the low-GALC activity referral samples from the New York State NBS program (2). Therefore, inheritance of either variant in its homozygous or compound heterozygous status is likely benign in the absence of p.I562T background (53).

### Human MMV cell model using psychosine levels as a readout

Psychosine may be a more accurate readout than residual GALC activity for GALC function, as it has a broader dynamic range and is more stable in clinical samples (43). Our results showed that it is feasible to establish human cell models with psychosine accumulation. Genetic KO of *GALC* induced psychosine accumulation in the oligodendrocytic MO3.13 cell line. Functional expression of GALC MMVs by transient transfection catabolized psychosine accumulated in the KO cells in an activity-dependent manner. The measured residual GALC activity in the MMV cell models were significantly correlated to the psychosine levels (Fig. 12*A*). However, the cellular psychosine levels did not correlate to the age of symptom onset in patients with these MMV genotypes (Fig. 12, *C* and *D*). The lack of clinical correlation with psychosine levels in the cell models is likely due to an insufficient penetrance of GALC expression in cell populations by transient transfection. In addition, the efficiency of cross-correction by secreted GALC may be low or absent in MMVs that severely impact GALC secretion. Therefore, technical strategies that can increase penetrance, such as increasing transfection efficiency and/or positive selection of GALC expressing cells, will likely improve the psychosine-clinical onset correlation in transient expression assay.

### Implications for clinical use and therapeutics

Genotype–phenotype correlations in KD have not been well established. In particular, the topic on functional deficiency caused by MMV genotypes has not been explored, although it is the most common type of pathogenic mutation. Our study highlights the importance of assessing residual GALC activity in combination with genotype analysis to better understand the clinical implications of GALC MMVs. The correlation between residual GALC activity determined in the *GALC*-KO cell model and the age of symptom onset provides a basis for using enzyme activity measurements as a tool for disease prognostication. Our plan is to expand the list of clinically relevant MMVs as more clinical information becomes available, which would facilitate more accurate prognostic assessments and inform management strategies for KD patients with MMV genotypes.

Our study also identifies MMVs that likely impair GALC function *via* a protein misfolding mechanism. Pharmacological chaperones (PCs) are small molecules that can act to restore protein folding by binding to the protein’s active site within the ER, thus stabilizing the misfolded proteins. 1-Deoxygalactonojirimycin, originally identified as a reversible, competitive inhibitor of α-galactosidase A, was the first PC approved by the Food and Drug Administration to treat patients with Fabry disease (54). For KD, PC candidates, such as iso-galacto-fagomine have been shown to bind GALC and increase thermal stability of the protein at nanomolar concentrations (55). Our current cell model and assays would be an ideal *in vitro* platform to screen and develop therapeutics, such as PCs for KD. GALC activity and psychosine levels could be considered functional readouts to determine potency, efficacy, and optimal dosing regimens. In addition, the platform would allow screening of mutations amenable to specific drugs, similar to the GLP-HEK assay used for Fabry disease (56).

In summary, our study provides a comprehensive analysis of the functional and trafficking effects of GALC MMVs in a *GALC*-KO cell model. The results offer valuable insights into the pathogenic mechanisms associated with the mutations and support the use of residual GALC activity as a tool to interpret pathogenicity of MMVs and establish genotype–phenotype correlations in KD. Continued research into the molecular mechanisms of GALC dysfunction will be essential for advancing our understanding of KD biology and developing MMV-specific therapeutic interventions.

## Experimental procedures

### MO3.13/*GALC*-KO cell line

MO3.13 is an immortalized human hybrid cell line created by fusion between primary oligodendrocyte and rhabdomyosarcoma. The cell line was purchased and authenticated by Cedarlane. The cells were tested for *mycoplasma* contamination by PCR-based method upon arrival and after the last experiment of the current study. It was found to be free of *mycoplasma* contamination throughout duration. Guided RNA targeting sequence 1 (sgRNA-GALC1: 5′ GAAAGCTATGACTGCGGCCG 3′) and 2 (sgRNA-GALC2: 5′ TACGTGCTCGACGACTCCGA 3′) were subcloned individually into pX459 v2.0 (gift from Dr Feng Zhang, Addgene plasmid #62988 (57)). GALC targeting sequences were further tested by the Off-Spotter software to prevent potential off target effects. Vectors were cotransfected into the MO3.13 cells, followed by puromycin selection (1 μg/ml) for 3 days. After 4 weeks of colony formation, multiple colonies were isolated and expanded for sequence validation. The sgRNAs were designed to specifically target 2 protospacer adjacent motif sites on exon 1 of the *GALC* gene. Positive clones with cleavage on the target sites created an 85-bp deletion and a downstream nonsense mutation at 319 nt of exon 2 (Fig. 3, *A* and *B*). We confirmed the absence of GALC protein (Fig. 3*C*) and GALC activity (Fig. 3*D*) in the *GALO*-KO cell line by WB and GALC activity assay, respectively. Substrate accumulation in the *GALC*-KO cell line was confirmed by psychosine analysis (Fig. 3*E*).

Proteomic analysis of native MO3.13 cells (WT) and MO3.13/*GALC*-KO cells (KO) using isobaric TMTs (Fig. 4*A*).

Total cell lysate (20 μg) from each sample was loaded onto the SDS-PAGE and run a few centimeters into the separating gel, which was stained with Coomassie blue. Whole lanes from individual samples were excised, minced, and subjected to standard trypsin digestion protocol with triethylammonium bicarbonate as the buffer. Digested peptides were dried and labeled with 10-plex TMT reagents (Thermo Fisher Scientific) according to the manufacturer’s protocols. Satisfactory labeling efficiency (>99%) was confirmed by mass spectrometry. Labeled peptides (50 μg) were combined and fractionated with high-pH reversed-phase spin columns (Thermo Fisher Scientific) into eight fractions. Fractions were dried and analyzed in technical duplicates by nanoflow ultra-performance liquid chromatography (EASY nLC) coupled to an Orbitrap Fusion mass spectrometer. A TMT-experiment specific instrument method with synchronous precursor selection and MS^3^ was used. Mass spectrometry data were analyzed using Proteome Discoverer 2.4, and product ion spectra were correlated to known sequences in SEQUEST. The false discovery rate was limited < 1% in the dataset. Normalization and quantification of TMT-labeled peptides and two-tailed *t* tests of nonnested design were performed in the Reporter Ions Quantifier node in Proteome Discoverer. Adjusted *p* values were calculated by the Benjamini-Hochberg procedure. A complete set of mass spectrometry and proteomics data files has been deposited in the ProteomeXchange Consortium *via* the PRIDE partner repository with the dataset identifier PXD057472. Detailed experimental proteomics methods can be found in Supplemental Information.

### Generation of *GALC* WT and mutant expression constructs

Full-length, human, WT *GALC* complementary DNA (SC120078, NM_000153) was originally purchased from Origene. The complementary DNA was subcloned into the pBApo-CMV pur expression vector at the XbaI and PstI sites (Takara Bio). V5 and 6-histidine tags were included on the carboxyl terminal for protein detection and purification purposes (24). Point mutations were introduced to the pBApo-GALC-VH construct with the Q5 site-directed mutagenesis kit (New England Biolabs). Nonoverlapping primer pairs for each MMV are listed in Table S1. Sanger DNA sequencing was performed on all clones to ensure no random mutations were introduced and that the desired mutation was present.

### Transient GALC variants expression study

GALC expression constructs were transfected to MO3.13/*GALC*-KO cells using Lipofectamine 3000 according to the manufacturer’s instructions (Thermo Fisher Scientific). Briefly, 500 ng of pBApo-GALC-VH plasmid was transfected to preplated cells on 12-well culture plates at 70%-80% density. The cells were cultured in completed medium (Dulbecco’s modified eagle medium with 10% (v/v) fetal bovine serum, and 1% (v/v) penicillin–streptomycin) for 72 h. To account for any variability in transfection efficiency, GALC activity and protein levels were normalized to the protein levels of puromycin N-acetyltransferase (PAC), which was expressed by the same DNA construct. Results presented in Table 3 are mean values ± SEM obtained from four independent experiments.

### Cell harvesting and sample preparation for analysis

At harvesting, culture medium was collected and centrifuged to remove floating cells and debris. The medium supernatant was saved for analysis of sec-GALC by ELISA. Cells were washed with cold PBS, and residual PBS was removed by vacuum suction. Cells were lysed using the M-Per mammalian extraction reagent supplemented with the 1x Halt protease inhibitor cocktail (Thermo Fisher Scientific) on ice for 20 min. The insoluble fraction was removed by centrifugation at 13,000 rcf for 30 min. Total soluble lysate was transferred to a clean tube and stored on ice. Protein concentration of lysate was measured by Pierce bicinchoninic acid protein assay (Thermo Fisher Scientific). Cell lysate and medium supernatant were kept on ice, but never frozen, prior to GALC activity, lys-GALC, pre-GALC, and sec-GALC analysis.

### *In vitro* GALC activity assay

Residual GALC enzymatic activity in cell lysate was measured by a fluorescence substrate turnover assay (58). Briefly, about 20 μg total protein was added to a reaction cocktail containing 0.1 M citric acid, 0.2 M sodium phosphate, 7 mg/ml sodium taurocholate, 2 mg/ml oleic acid, and 0.5 mg/ml 6-hexadecanoylamino-4-methylumbelliferyl-β-D-galactopyranoside (HMGal; Biosynth Inc) at pH 4.5. The reaction was incubated for 4 h at 37 °C and stopped by adding 2 volumes of 0.1 M glycine/0.1 M sodium hydroxide solution and 4 volumes of absolute ethanol. The concentration of reaction product was determined by fluorescence plate reader (ex 385 nm/em 450 nm). A 4-methylumbelliferone standard curve was ran in parallel for calculation of absolute GALC activity unit expressed in nmol per hour per milligram protein.

### Western blot

Cultured cells were lysed in M-Per lysis buffer (Thermo Fisher Scientific) supplemented with protease inhibitors. After centrifugation, the protein concentration of the supernatant was determined by bicinchoninic acid assay for normalization, and 30 μg of total protein was resolved by SDS-PAGE with 10 to 20% Tris-glycine gel. Gel proteins were transferred to polyvinylidene fluoride membranes by Criterion blotter (Bio-Rad) according to manufacturer’s instructions. The protein blot was blocked in 5% nonfat milk for 1 h at room temperature, followed by incubation with primary antibody for 16 to 18 h at 4 °C. Blots were probed with horseradish peroxidase (HRP)-conjugated secondary antibody for 1 h at room temperature, followed by signal development using the Immobilon Western Chemiluminescent HRP substrate (MilliporeSigma) following manufacturer’s instructions. WB images were obtained using the ImageQuant LAS-4000 imager (GE Healthcare Life Sciences). Primary antibodies for WB analysis include anti-GALC (CL13.1, 1:4000) (59), anti-PAC (1:2,000, Thermo Fisher Scientific) and anti-GAPDH (1:100,000, Proteintech). Specificity of antibodies was validated either by knockout cells (GALC) and/or ectopic overexpression experiment (GALC and PAC). For blot quantification, protein levels of lys-GALC (Fig. 7) and pre-GALC (Fig. 9*A*) were analyzed by densitometric measurement of target band intensity normalized to GAPDH levels using the ImageJ software. After that the protein levels were also normalized to PAC levels to adjust for transfection efficiency.

### GALC enzyme-linked immunosorbent assay (ELISA)

Secretory GALC levels were measured using 50 μl of culture supernatant added to a Nunc Maxisorp 96-well plate precoated with 2 μg/ml anti-V5 tag antibody and preblocked in complete growth medium (DMEM with 10% feta bovine serum). Standard curve samples were prepared by diluting recombinant human GALC protein (R&D Systems) in culture supernatant from KO cells to 0.8, 1.6, 3.2, 6.3, 12.5, 25, and 50 ng/ml. Samples and standards were incubated for 2 h. Anti-GALC antibody, CL13.1, followed by anti-mouse IgG1-HRP secondary antibody, both diluted in sample buffer (PBS with 1% bovine serum albumin) to 0.5 μg/ml, were added to plates and incubated for an hour. 3,3′,5,5′-Tetramethylbenzidine substrate was added and incubated for 2 min. Diluted sulfuric acid (0.16 M) was used to stop the reactions. Plates were read on the SpectraMax Plus 384 microplate reader (450 nm). All procedures except antibody coating were performed at room temperature. Plates were washed between blocking, sample addition and antibody addition using PBS with 0.05% Tween-20. GALC protein concentration was calculated by interpolation of absorbance values to a normalized standard curve.

Intracellular pre-GALC levels were measured as described above, except 30 μg of protein from total lysate was added to precoated ELISA plates and preblocked in sample buffer. Standard curve samples were prepared by diluting recombinant GALC protein in sample buffer, supplemented with 30 μg of lysate protein from the *GALC*-KO cells.

### Psychosine measurements

Cultured cells were washed and scrapped into cold PBS. Cell pellets were collected by centrifugation at 13,000 rcf for 10 min and stored at −80 °C. Replicate sets of cell pellets were lysed in M-Per lysis buffer to determine the total protein concentration and psychosine level in pico-mole per milligrams protein. For psychosine analysis in the correlation study (Fig. 12), cultured cells were washed in cold PBS then lysed by digitonin (0.1%) containing Tris-buffered saline supplemented with 1x Halt protease inhibitor cocktail. After centrifugation, the insoluble lipid pellet was saved and stored at −80 °C for analysis. Soluble lysate was analyzed for total protein concentration and residual GALC activity.

Frozen samples were sent to Gelb’s lab for psychosine analysis. Briefly, cell pellets were homogenized with 50 μl of 0.9% sodium chloride solution. Internal standard solution (250 μl, 1 nM d5-psychosine) was added and incubated with the sample for 2 h. The mixture was centrifuged at 13,000 rcf for 5 min. Supernatant went through solid-phase extraction cleanup and was dried by speedvac. The sample was reconstituted with 100 ml of mobile phase B solution, followed by injection to the MedChem Xevo TQS system. Detailed procedures for UPLC-MS/MS analysis were recently described (60).

### Statistical analysis

Figure 3: Statistical differences in GALC activity (n = 4, independent experimental replicates) or psychosine level (n = 3, independent experimental replicates) between WT and GALC-KO cells were analyzed by a two-tailed, unpaired *t* test at 95% confidence interval. (∗∗*p* < 0.01, ∗∗∗*p* < 0.001).

Figure 5: Statistical differences in GALC activity between individual MMV (n = 4, independent experimental replicates) and WT-GALC (n = 24, independent experimental replicates) were analyzed by a two-tailed, unpaired *t* test at 95% confidence interval. (# - no significant difference, ∗*p* < 0.05, ∗∗*p* < 0.01, ∗∗∗*p* < 0.001, no label - *p* < 0.0001). Figures 8*A* and 9*C*: Statistical differences in sec-GALC levels (n = 4) or pre-GALC levels (n = 3) among cells expressing individual MMVs versus WT were analyzed by a two-tailed, unpaired *t* test at 95% confidence interval. (n = 4, independent experimental replicates, ∗*p* < 0.05, ∗∗*p* < 0.01, ∗∗∗*p* < 0.001, ∗∗∗*p* < 0.0001).

Figure 10: Statistical differences in (A) GALC activity, (B) sec-GALC level and (C) pre-GALC levels between cells expressing the p.562I MMV and the same MMV on the p.562T background were analyzed by a two-tailed, unpaired *t* test at 95% confidence interval. (n = 3–4, independent experimental replicates, ∗*p* < 0.05, ∗∗*p* < 0.01, ∗∗∗*p* < 0.001, ∗∗∗∗*p* < 0.0001). Tables 3 and 4: Statistical differences between cells expressing individual MMVs and WT, between cells expressing MMVs on p.562T and the same MMV on the p.562I background, were analyzed by a two-tailed, unpaired *t* test at 95% confidence interval. (∗*p* < 0.05, ∗∗*p* < 0.01, ∗∗∗*p* < 0.001, ∗∗∗∗*p* < 0.0001).

Figures 6, *B* and *C*, 7*E*, 8, *B* and *C*, 9*B*, 10, *D* and *E*, 11, *A*–*C*, 12, *A*–*D*: Correlation between groups was analyzed by Pearson correlation test. Significance of the Pearson correlation coefficient (*i.e.*, Pearson r) was determined by a *p* value at 95% confidence interval. (∗*p* < 0.05, ∗∗*p* < 0.01, ∗∗∗*p* < 0.001, ∗∗∗∗*p* < 0.0001).

## Data availability

All data described in this manuscript are contained within the manuscript except for the dataset related to the proteomics study. A complete set of mass spectrometry and proteomics data files has been deposited to the ProteomeXchange Consortium *via* the PRIDE partner repository with the dataset identifier PXD057472.

## Supporting information

This article contains supporting information.

## Conflict of interest

The authors declare that they have no conflicts of interest with the contents of this article.
